# Advanced oxidation protein products attenuate the autophagy-lysosome pathway in ovarian granulosa cells by modulating the ROS-dependent mTOR-TFEB pathway

**DOI:** 10.1038/s41419-024-06540-w

**Published:** 2024-02-21

**Authors:** Xing-Yu Zhou, Yun-Hui Lai, Jun Zhang, Ying Li, Xiao-Min Wu, Yi-Zhen Yang, Xiao-Fei Zhang, Lin-Zi Ma, Ke-Ming Zheng, Yu-Dong Liu, Zhe Wang, Shi-Ling Chen

**Affiliations:** Center for Reproductive Medicine, Department of Gynecology and Obstetrics, Nanfang Hospital, Southern Medical University, Guangzhou, PR China

**Keywords:** Endocrine reproductive disorders, Endocrine reproductive disorders

## Abstract

Oxidative stress dysfunction has recently been found to be involved in the pathogenesis of premature ovarian insufficiency (POI). Previously, we found that advanced oxidation protein products (AOPPs) in plasma were elevated in women with POI and had an adverse effect on granulosa cell proliferation. However, the mechanism underlying the effects of AOPPs on autophagy-lysosome pathway regulation in granulosa cells remains unclear. In this study, the effect of AOPPs on autophagy and lysosomal biogenesis and the underlying mechanisms were explored by a series of in vitro experiments in KGN and COV434 cell lines. AOPP-treated rat models were employed to determine the negative effect of AOPPs on the autophagy-lysosome systems in vivo. We found that increased AOPP levels activated the mammalian target of rapamycin (mTOR) pathway, and inhibited the autophagic response and lysosomal biogenesis in KGN and COV434 cells. Furthermore, scavenging of reactive oxygen species (ROS) with N-acetylcysteine and blockade of the mTOR pathway with rapamycin or via starvation alleviated the AOPP-induced inhibitory effects on autophagy and lysosomal biogenesis, suggesting that these effects of AOPPs are ROS-mTOR dependent. The protein expression and nuclear translocation of transcription factor EB (TFEB), the key regulator of lysosomal and autophagic function, were also impaired by the AOPP-activated ROS-mTOR pathway. In addition, TFEB overexpression attenuated the AOPP-induced impairment of autophagic flux and lysosomal biogenesis in KGN and COV434 cells. Chronic AOPP stimulation in vivo also impaired autophagy and lysosomal biogenesis in granulosa cells of rat ovaries. The results highlight that AOPPs lead to impairment of autophagic flux and lysosomal biogenesis via ROS-mTOR-TFEB signaling in granulosa cells and participate in the pathogenesis of POI.

## Introduction

Premature ovarian insufficiency (POI) is defined as exhaustion of ovarian reserve before the age of 40 and is an incurable cause of infertility in women of childbearing age [1]. POI is characterized by menstrual disturbance (amenorrhea or oligomenorrhea) accompanied by increased gonadotropin levels and decreased estradiol levels [2]. Approximately 1% of women under 40 years old suffer from POI, and its etiology manifests with high heterogeneity. Although studies have suggested that the causes of POI include genetic mutations, immune factors, and iatrogenic factors (chemotherapy, radiotherapy and ovarian surgery), the causes of most cases are still unclear [3].

Recent studies have revealed that excessive oxidative stress plays a key role in the etiology of POI [4]. As protein biomarkers of oxidative stress, advanced oxidation protein products (AOPPs) have recently attracted considerable attention due to a series of studies demonstrating their accumulation in patients with numerous chronic inflammatory diseases, including chronic kidney disease [5], endometriosis [6], and osteoporosis [7], etc. Our latest study found that the plasma AOPP levels in both women with POI and women with biochemical POI were significantly higher than those in controls, and plasma AOPP levels were negatively correlated with anti-Müllerian hormone levels and antral follicle counts. The study also highlighted that elevated AOPP levels induced G1/G0-phase arrest by activating the reactive oxygen species (ROS)- c-Jun N-terminal kinase (JNK)/p38 mitogen-activated protein kinase (MAPK) -p21 signaling pathway in ovarian granulosa cells, thus contributing to the pathogenesis of POI [8]. However, previous studies have found AOPPs, as oxidative mediators, implicated in a variety of pathological changes and multiple pathogenic mechanisms, and the other effects of AOPPs on granulosa cells still warrant further investigation.

The autophagy-lysosome pathway is an evolutionarily conserved process in eukaryotic cells that involves the transport of damaged organelles and proteins in autophagosomes to lysosomes, which form autolysosomes for degradation [9]. The autophagy-lysosome pathway is critical in maintaining cellular homeostasis under stress conditions, and repressed autophagy is observed in various diseases, including POI [10–12]. Recent studies have indicated that AOPPs can inhibit autophagy in renal tubular epithelial cells [13] and induce lysosomal dysfunction in macrophages [14], but the effect of AOPPs on the autophagy-lysosome pathway in granulosa cells is still unclear. Moreover, lysosomes are indispensable organelles in the autophagy-lysosome pathway [15], but whether impaired lysosomal biogenesis in ovarian granulosa cells participates in the etiology of POI has never been reported. Transcription factor EB (TFEB), an important transcription factor that regulates autophagic and lysosomal function, is regulated by the mammalian target of rapamycin (mTOR) pathway, a classic autophagy-related pathway [16]. However, few studies, to the best of our knowledge, have demonstrated changes in TFEB expression and subcellular localization and the probability of autophagy alteration by TFEB in granulosa cells.

In the present study, we investigated the molecular mechanism by which AOPPs impair autophagy and lysosome biogenesis in ovarian granulosa cells, aiming to further clarify the mechanism of AOPPs in the pathogenesis of POI. We found that AOPP-induced intracellular ROS accumulation regulated the mTOR-TFEB signaling pathway, thus impairing autophagy and lysosomal biogenesis both in vitro and in vivo. AOPP-stimulated damage to the autophagy-lysosome pathway results in granulosa cell dysfunction, eventually participating in the occurrence and development of POI.

## Results

### AOPPs inhibited autophagy and lysosomal biogenesis in granulosa cells

**Fig. 1:**
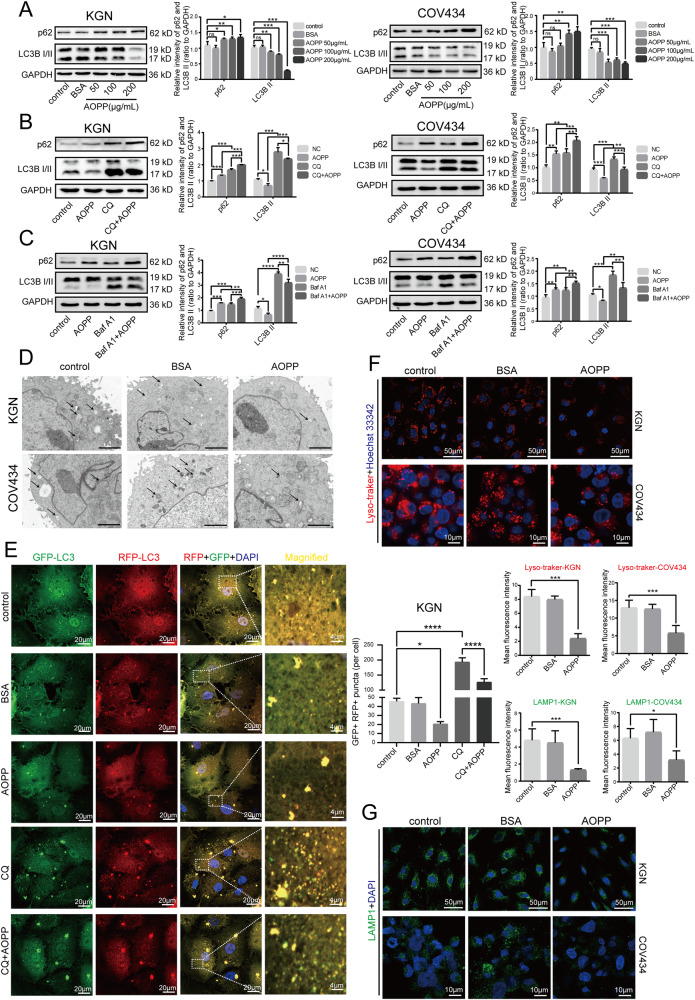
AOPP inhibited autophagy and lysosomal biogenesis in granulosa cells. **A** Representative blots and quantification showing p62 and LC3B II expression levels in negative control and KGN/COV434 cells treated with 200 μg/mL BSA, 50 μg/mL AOPPs, 100 μg/mL AOPPs, and 200 μg/mL AOPPs for 48 h. **B** Representative blots and quantification showing p62 and LC3B II expression levels in negative control, KGN/COV434 cells treated with 200 μg/mL AOPPs for 48 h (AOPP group), KGN/COV434 cells treated with 30 μM CQ for 4 h (CQ group), and KGN/COV434 cells pretreated with AOPPs for 48 h and then treated with CQ for 4 h (CQ + AOPP group). **C** Representative blots and quantification showing p62 and LC3B II expression levels in negative control, KGN/COV434 cells treated with 200 μg/mL AOPPs for 48 h (AOPP group), KGN/COV434 cells treated with 100 nM nM Baf A1 for 4 h (Baf A1 group), and KGN/COV434 cells pretreated with AOPPs for 48 h and then treated with Baf A1 for 4 h (Baf A1 + AOPP group). **D** Ultrastructural analysis of autophagosomes in negative control and KGN/COV434 cells treated with 200 μg/mL BSA (the BSA group) and 200 μg/mL AOPPs for 48 h (the AOPP group). The arrows indicate autophagosomes. Scale bars: 2 μm. **E** Representative images of RFP-GFP-LC3 under a confocal laser scanning microscope in control group, BSA group, AOPP group, CQ group, and CQ + AOPP group. The GFP + RFP+ (yellow) puncta are autophagosomes. The numbers of autophagosomes were quantified for 10 cells. High-magnification images of the indicated boxed areas are shown in the rightmost panels. **F** Lysosomal biogenesis assessed by Lyso-Tracker Red in control group, BSA group, and AOPP group. **G** LAMP1 expression as detected by immunofluorescence in control group, BSA group, and AOPP group. All experiments were repeated for three times. The results are expressed as the mean ± SD from experiments performed in triplicate. *P < 0.05, **P < 0.01, ***P < 0.001, ****P < 0.001. Statistical significance was determined by one-way ANOVA and Tukey tests for post hoc comparisons.

The autophagy-lysosome pathway is a highly regulated and evolutionarily conserved catabolic process that enables cells to remove and recycle intracytoplasmic material during times of stress or starvation [17]. To confirm whether autophagy and lysosomal biogenesis were involved in the effects of AOPPs on KGN and COV434 cells, we evaluated proteins associated with autophagic flux, including microtubule associated protein 1 light chain 3BII (LC3B II) and p62 (also known as sequestosome 1, SQSTM1) [18, 19], and the numbers of autophagosomes and lysosomes in cultured KGN and COV434 cells treated with increasing concentrations of AOPPs and 200 μg/mL bovine serum albumin (BSA). We observed an evident concentration-dependent decrease in the LC3B II protein levels in KGN and COV434 cells that were treated with 50, 100 and 200 μg/mL AOPP for 48 h, while the levels of p62 showed the opposite trend, suggesting that autophagic flux was impaired in the AOPP-treated KGN and COV434 cells (Fig. 1A). To confirm the impairment of autophagic flux, KGN cells were treated with or without chloroquine (CQ), an autophagy inhibitor that causes lysosome dysfunction, in the presence or absence of AOPPs. We found that CQ alone increased endogenous LC3B II and p62 expression levels, which reflect basal autophagy in KGN and COV434 cells, while CQ in combination with AOPPs treatment decreased LC3B II expression and further increased p62 expression, confirming that AOPPs inhibited autophagic flux in KGN and COV434 cells (Fig. 1B). Bafilomycin A1 (Baf A1) could also inhibit acidification in lysosomes, resulting in the blockade of autophagosome-lysosome fusion. A similar trend in LC3B II and p62 expression levels was observed in both KGN and COV434 cells, after treatment with CQ or Baf A1 (Fig. 1C). Collectively, based on decreased LC3B II and increased p62 levels, the data indicated that AOPPs may induce insufficient autophagy in ovarian granulosa cells.

The transmission electron microscopy results also showed that the number of autophagosomes was lower after AOPP stimulation than it in the control group or after BSA treatment (Fig. 1D). To further visualize the inhibitory effect of AOPPs on autophagy, KGN cells were transiently transfected with tandem red fluorescence protein (RFP) - green fluorescence protein (GFP)-LC3 adenovirus, in which LC3 was tagged with acid-sensitive GFP and acid-resistant monomeric RFP, to measure the formation of autophagosomes (GFP + RFP+ puncta) and autolysosomes (RFP+ puncta). Our results showed that, compared with the control and BSA treatment conditions, treatment of KGN cells with 200 μg/ml AOPP significantly decreased autophagosome numbers, whereas treatment with 30 μM CQ increased autophagosome numbers. Furthermore, the number of autophagosomes was significantly greater in KGN cells cotreated with AOPPs and CQ than in KGN cells treated with CQ alone, further verifying that AOPP stimulation impaired autophagy in KGN cells (Fig. 1E).

Hence, to further investigate the mechanisms underlying AOPP-induced autophagy, we evaluated changes in lysosomal biogenesis using a lysosomal probe (Lyso-Tracker) and detected the expression levels of lysosomal-associated membrane protein 1 (LAMP1), which can be used as a marker of lysosomal generation. The results showed that the number of lysosomes detected by Lyso-Tracker was lower in the AOPP group than in the control and BSA groups (Fig. 1F). LAMP1 expression showed a trend consistent with the number of lysosomes, indicating that AOPPs inhibited lysosomal biogenesis in KGN and COV434 cells (Fig. 1G). Taken together, these results indicated that AOPP treatment inhibited autophagy and lysosomal biogenesis in KGN and COV434 cells.

### NAC pretreatment alleviated the AOPP-induced inhibitory effects on autophagy and lysosomal biogenesis in granulosa cells

**Fig. 2:**
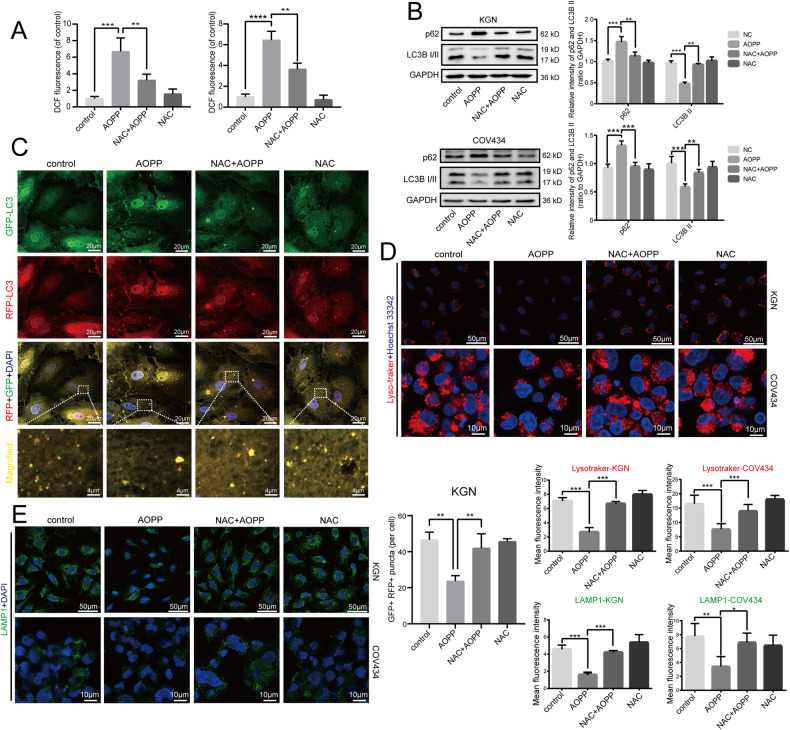
NAC pretreatment alleviated the AOPP-induced inhibitory effects on autophagy and lysosomal biogenesis in granulosa cells. **A** A DCF fluorescence assay showing the intracellular ROS production in negative control, KGN/COV434 cells treated with 200 μg/mL AOPPs for 48 h (the AOPP group), cells treated with 2 mM NAC for 49 h (the NAC group), and cells pretreated with 2 mM NAC for 1 h and then treated with AOPPs for 48 h (the NAC + AOPP group). **B** Representative blots and quantification showing p62 and LC3B II expression levels in the control group, AOPP group, NAC group, and NAC + AOPP group. **C** Representative images of RFP-GFP-LC3 under a confocal laser scanning microscope in the control group, AOPP group, NAC group, and NAC + AOPP group. The GFP + RFP+ (yellow) puncta are autophagosomes. The numbers of autophagosomes were quantified for 10 cells. High-magnification images of the indicated boxed areas are shown in the bottom panels. **D** Lysosomal biogenesis assessed by Lyso-Tracker Red in the control group, AOPP group, NAC group, and NAC + AOPP group. **E** LAMP1 expression as detected by immunofluorescence in the control group, AOPP group, NAC group, and NAC + AOPP group. All experiments were repeated for three times. The results are expressed as the mean ± SD from experiments performed in triplicate. *P < 0.05, **P < 0.01, ***P < 0.001. Statistical significance was determined by one-way ANOVA and Tukey tests for post hoc comparisons.

In our previous research, we found that AOPPs induced G1/G0-phase arrest in KGN cells; this effect relied on intracellular ROS generation and was concentration- and time-dependent [8]. Therefore, we sought to investigate whether the inhibitory effects of AOPPs on autophagy and lysosomal biogenesis in the present study were also mediated by ROS. The results of the dichlorofluorescein (DCF) fluorescence assay showed that in both KGN cells and COV434 cells, treatment with NAC could significantly decreased the levels of intracellular ROS (Fig. 2A).

We pretreated KGN and COV434 cells with 2 mM NAC, a ROS scavenger, for 1 h prior to AOPP exposure and then treated cells with both 2 mM NAC and 200 μg/ml AOPP for 48 h to evaluate whether ROS were involved in AOPP-induced autophagy inhibition. As shown in Fig. 2B, western blots showed that the increase in p62 protein expression and the reduction in LC3B II protein expression caused by AOPP challenge were both abolished by NAC pretreatment. Autophagic flux in KGN cells was further determined through an RFP-GFP-LC3 transfection assay. A significant increase in the number of autophagosomes was observed upon cotreatment with NAC and AOPPs, which indicated that the AOPP-induced inhibitory effect on autophagy was alleviated by NAC (Fig. 2C), suggesting that the accumulation of ROS contributed to AOPP-induced autophagy inhibition in granulosa cells.

We further investigated whether the inhibition of lysosomal biogenesis induced by AOPPs could also be reversed by NAC administration. The Lyso-Tracker results showed that the reduction in the number of intracellular lysosomes after AOPP treatment was remarkably attenuated by NAC pretreatment (Fig. 2D). Similarly, the LAMP1 expression level in the NAC + AOPP group was significantly higher than that in the AOPP group (Fig. 2E). Therefore, our results indicated that the inhibition of autophagy and lysosomal biogenesis by AOPPs was mediated by ROS accumulation and suggested that eliminating intracellular ROS may block the suppression of autophagy and lysosomal biogenesis induced by AOPPs.

### AOPPs inhibited autophagy and lysosomal biogenesis via the ROS-dependent mTOR pathway in granulosa cells

**Fig. 3:**
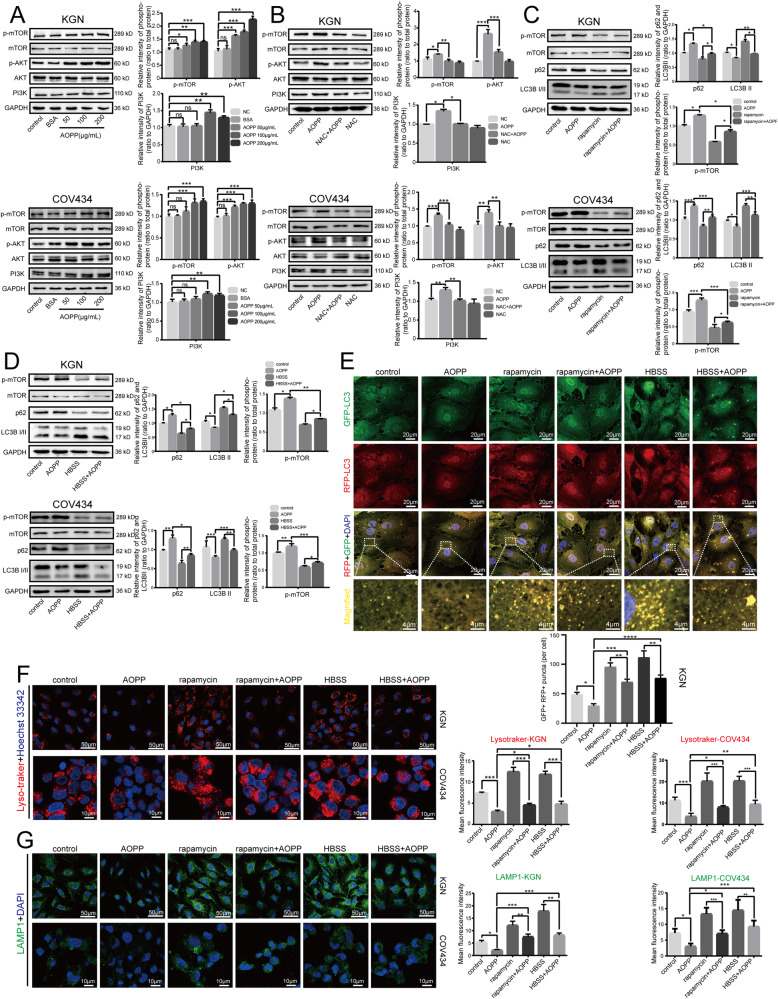
AOPPs inhibited autophagy and lysosomal biogenesis via the ROS-dependent mTOR pathway in granulosa cells. **A** Representative blots and quantification showing mTOR, p-mTOR, AKT, p-AKT and PI3K expression levels in negative control cells and KGN/COV434 cells treated with 200 μg/mL BSA, 50 μg/mL AOPPs, 100 μg/mL AOPPs, and 200 μg/mL AOPPs for 48 h. **B** Representative blots and quantification showing p-mTOR, p-AKT and PI3K expression levels in the control group, AOPP group, NAC group, and NAC + AOPP group. **C** Representative blots and quantification showing p-mTOR, p-AKT and PI3K expression levels in negative control cells, KGN/COV434 cells treated with 200 μg/mL AOPPs for 48 h (AOPP group), KGN/COV434 cells treated with 1 μM rapamycin for 48 h (rapamycin group), and KGN/COV434 cells pretreated with 1 μM rapamycin for 1 h and then treated with AOPPs for 48 h (rapamycin + AOPP group). **D** Representative blots and quantification showing p-mTOR, p-AKT and PI3K expression levels in negative control cells, KGN/COV434 cells treated with 200 μg/mL AOPPs for 48 h (AOPP group), cells starved in HBSS for 3 h for KGN cells and 1 h for COV434 cells (HBSS group), and cells treated with AOPPs for 48 h and then starved with HBSS for 3 h for KGN cells and 1 h for COV434 cells (HBSS + AOPP group). **E** Representative images of RFP-GFP-LC3 under a confocal laser scanning microscope in the control group, AOPP group, rapamycin group, rapamycin + AOPP group, HBSS group and HBSS + AOPP group. The GFP + RFP+ (yellow) puncta are autophagosomes. The numbers of autophagosomes were quantified for 10 cells. High-magnification images of the indicated boxed areas are shown in the bottom panels. **F** Lysosomal biogenesis assessed by Lyso-Tracker Red in the control group, AOPP group, rapamycin group, rapamycin + AOPP group, HBSS group and HBSS + AOPP group. **G** LAMP1 expression as detected by immunofluorescence in the control group, AOPP group, rapamycin group, rapamycin + AOPP group, HBSS group and HBSS + AOPP group. All experiments were repeated for three times. The results are expressed as the mean ± SD from experiments performed in triplicate. *P < 0.05, **P < 0.01, ***P < 0.001, ****P < 0.0001. Statistical significance was determined by one-way ANOVA and Tukey tests for post hoc comparisons.

The mTOR pathway is a well-known autophagy-regulated pathway [20] that also plays important roles in folliculogenesis in the ovaries [21]. To verify whether AOPP-induced autophagy inhibition in granulosa cells was mediated by the mTOR pathway, we assessed the expression levels of mTOR and the upstream proteins phosphatidylinositol-3-kinase (PI3K) and serine/threonine protein kinase (AKT/PKB) by western blot analysis. The results showed that the phosphorylation of AKT and mTOR as well as the expression of PI3K protein were all increased in KGN and COV434 cells treated with elevated AOPP concentrations (Fig. 3A) and that NAC cotreatment inhibited the AOPP-induced upregulation of AKT, mTOR phosphorylation and PI3K expression (Fig. 3B). These findings suggested the involvement of the ROS-dependent mTOR pathway in AOPP-induced autophagy impairment.

To further supported the above observations, cells were treated with or without selective inhibitors of mTOR (rapamycin) or Hanks’ balanced salt solution (HBSS) for amino acid starvation to inhibit mTOR phosphorylation. We found that treatment with rapamycin or HBSS significantly inhibited mTOR phosphorylation in KGN cells. Compared with treatment with AOPPs alone, AOPPs in combination with either rapamycin or HBSS further decreased mTOR phosphorylation and p62 expression and increased LC3B II expression, suggesting that inhibition of mTOR reversed the impairment of autophagic flux induced by AOPPs (Fig. 3C, D). The RFP-GFP-LC3 adenovirus transfection results were also consistent with the western blot results, as rapamycin or HBSS administration significantly attenuated AOPP-induced autophagy inhibition, as demonstrated by the increased number of autophagosomes (Fig. 3E). Furthermore, we tested whether blocking the mTOR pathway could reverse the AOPP-induced inhibition of lysosomal biogenesis. The Lyso-Tracker results showed that the number of lysosomes was significantly increased after rapamycin or HBSS treatment. In addition, rapamycin or HBSS administration combined with AOPP treatment resulted in significantly more lysosomes than AOPP treatment alone (Fig. 3F). Furthermore, the reduction in LAMP1 fluorescence intensity in the AOPP group observed via immunofluorescence staining was prevented by rapamycin or HBSS (Fig. 3G). Taken together, these data provide strong evidence that the ROS-mTOR signaling cascade mediates the inhibitory effects of AOPPs on autophagy and lysosomal biogenesis and suggest that blocking the mTOR pathway is a promising strategy to attenuate the autophagy and lysosomal biogenesis disorders induced by AOPPs.

### AOPPs suppressed the expression and nuclear translocation of TFEB in granulosa cells via the ROS-mTOR pathway

**Fig. 4:**
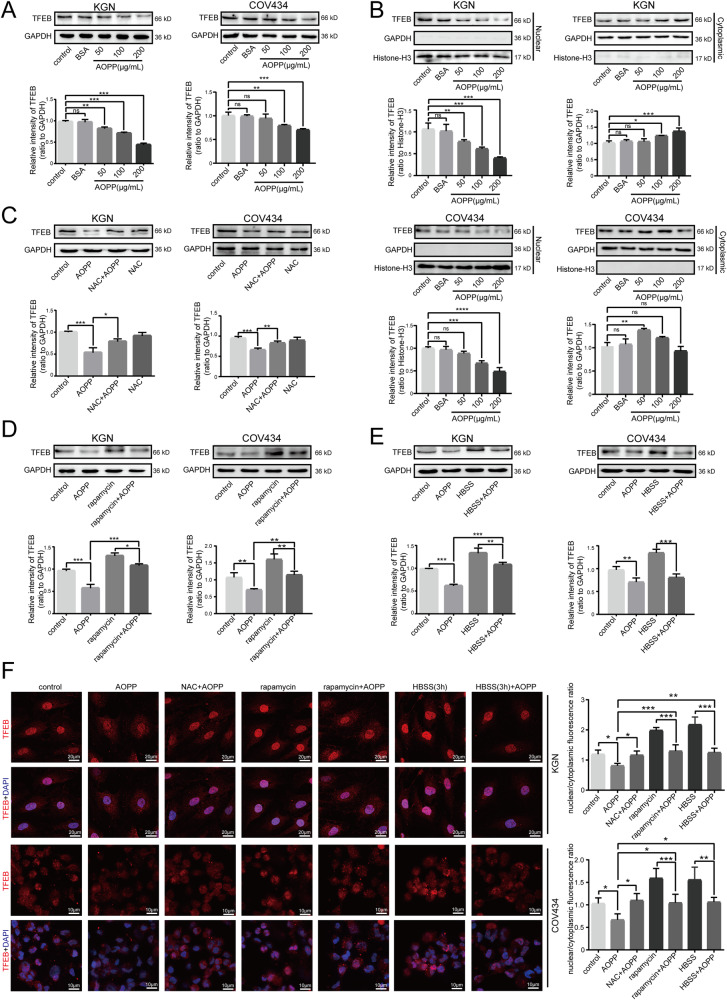
AOPP suppressed the expression and nuclear translocation of TFEB in granulosa cells via the ROS-mTOR pathway. **A** Representative blots and quantification showing TFEB expression levels in negative control cells and KGN/COV434 cells treated with 200 μg/mL BSA, 50 μg/mL AOPPs, 100 μg/mL AOPPs, and 200 μg/mL AOPPs for 48 h. **B** Representative blots and quantification showing TFEB in the nuclear and cytoplasmic fractions in negative control cells and KGN/COV434 cells treated with 200 μg/mL BSA, 50 μg/mL AOPPs, 100 μg/mL AOPPs, and 200 μg/mL AOPPs for 48 h. **C** Representative blots and quantification showing TFEB expression levels in the control group, AOPP group, NAC group, and NAC + AOPP group. **D** Representative blots and quantification showing TFEB expression levels in the control group, AOPP group, rapamycin group, and rapamycin + AOPP group. **E** Representative blots and quantification showing TFEB expression levels in the control group, AOPP group, HBSS group and HBSS + AOPP group. **F** Immunofluorescence staining of TFEB in the control group, AOPP group, NAC + AOPP group, rapamycin group, rapamycin + AOPP group, HBSS group and HBSS + AOPP group. All experiments were repeated for three times. The results are expressed as the mean ± SD from experiments performed in triplicate. *P < 0.05, **P < 0.01, ***P < 0.001. Statistical significance was determined by one-way ANOVA and Tukey tests for post hoc comparisons.

TFEB, a major regulator of autophagy and lysosomal biogenesis, has been found to regulate nearly two-thirds of genes encoding lysosomal biogenesis and autophagy proteins, thus maintaining the balance of the autophagy-lysosome pathway [16]. As a nuclear transcription factor, TFEB also shuttles from the cytoplasm into the nucleus to regulate downstream genes after being activated. To explore the potential role of TFEB in AOPP-induced autophagy inhibition, we assessed the expression and intracellular localization of TFEB following AOPP treatment. The results showed that AOPP challenge significantly inhibited TFEB expression in a concentration-dependent manner, indicating an essential role of TFEB in autophagic flux disruption (Fig. 4A). As shown in Fig. 4B, AOPPs markedly reduced nuclear TFEB protein levels in a concentration-dependent manner. The results of immunofluorescence staining revealed decreased nuclear staining of TFEB and simultaneously increased cytoplasmic staining in treated cells, indicating that TFEB nuclear translocation was disrupted following AOPP challenge (Fig. 4F).

To verify whether TFEB is a downstream molecule of the ROS-mTOR pathway activated by AOPPs, we tested the effects of treatment with the ROS scavenger NAC, treatment with the mTOR inhibitor rapamycin and starvation with HBSS on TFEB expression. We found that the reduction in TFEB expression was significantly attenuated by NAC (Fig. 4C), rapamycin (Fig. 4D) or HBSS starvation (Fig. 4E) cotreatment, suggesting that the ROS-mTOR pathway is critical for AOPPs to inactivate TFEB expression.

To further confirm whether the AOPP-induced inhibition of TFEB nuclear translocation was mediated by the ROS-mTOR pathway, NAC treatment, rapamycin treatment or HBSS starvation was conducted before AOPP treatment. We found that inhibition of ROS-mTOR signaling with NAC treatment, rapamycin treatment or HBSS starvation restored TFEB expression and facilitated TFEB shuttling from the cytoplasm into the nucleus (Fig. 4F). Taken together, these data indicated that AOPPs reduced the expression and nuclear translocation of TFEB in KGN and COV434 cells mainly via the ROS-mTOR pathway.

### Overexpression of TFEB alleviated the AOPP-induced inhibitory effects on autophagy and lysosomal biogenesis in granulosa cells

**Fig. 5:**
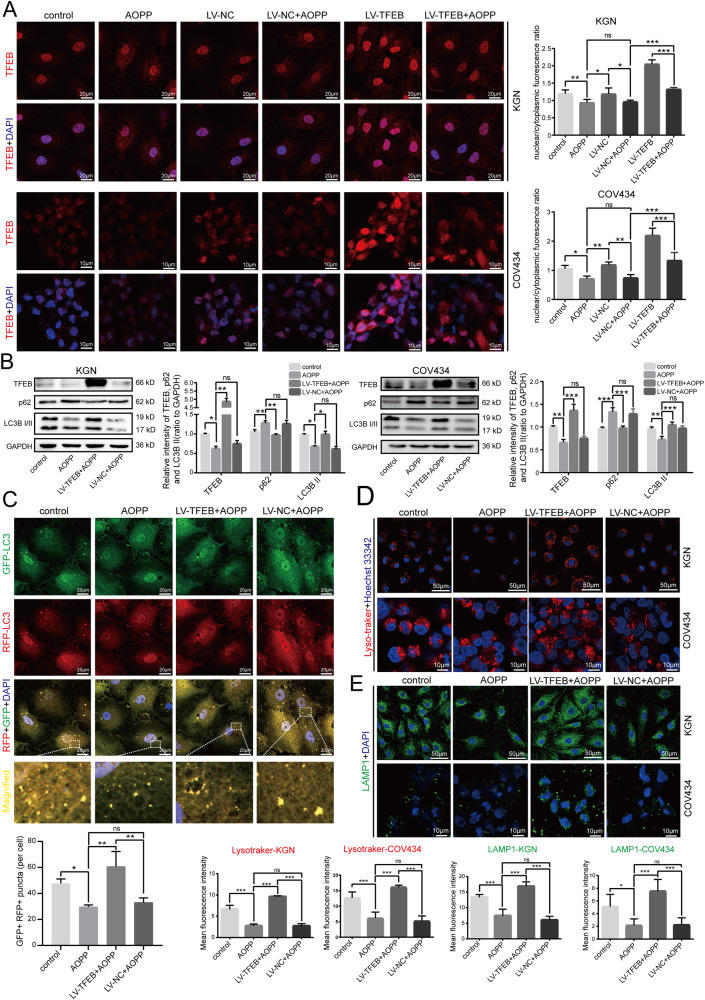
Overexpression of TFEB alleviated the AOPP-induced inhibitory effects on autophagy and lysosomal biogenesis in granulosa cells. **A** Immunofluorescence staining of TFEB in negative control cells, KGN/COV434 cells treated with 200 μg/mL AOPPs for 48 h (AOPP group), cells transfected with LV-NC (LV-NC group), cells transfected with LV-NC and treated with AOPPs (LV-NC + AOPP group), cells transfected with LV-TFEB (LV-TFEB group), and cells transfected with LV-TFEB and treated with AOPPs (LV-TFEB + AOPP group). **B** Representative blots and quantification showing p62 and LC3B II expression levels in the control group, AOPP group, LV-TFEB + AOPP group and LV-NC + AOPP group. **C** Representative images of RFP-GFP-LC3 under a confocal laser scanning microscope in KGN cells in the control group, AOPP group, LV-TFEB + AOPP group and LV-NC + AOPP group. The GFP + RFP+ (yellow) puncta are autophagosomes. The numbers of autophagosomes were quantified for 10 cells. High-magnification images of the indicated boxed areas are shown in the bottom panels. **D** Lysosomal biogenesis assessed by LysoTracker Red in the control group, AOPP group, LV-TFEB + AOPP group and LV-NC + AOPP group. **E** LAMP1 expression as detected by immunofluorescence in the control group, AOPP group, LV-TFEB + AOPP group and LV-NC + AOPP group. All experiments were repeated for three times. The results are expressed as the mean ± SD from experiments performed in triplicate. *P < 0.05, **P < 0.01, ***P < 0.001. Statistical significance was determined by one-way ANOVA and Tukey tests for post hoc comparisons.

To further verify the hypothesis that inhibition of autophagy and lysosomal synthesis in KGN and COV434 cells by AOPPs was mediated by TFEB, LV-TFEB and LV-NC were constructed, and further analyses were performed. Our above experiments showed that AOPPs can inhibit the translocation of TFEB to the nucleus. To verify whether LV-TFEB can promote the translocation of TFEB to the nucleus, immunofluorescence experiments were conducted to assess the nuclear and cytoplasmic distribution of TFEB. As illustrated in Fig. 5A, LV-TFEB significantly increased the expression of TFEB in the nuclei of KGN and COV434 cells, while LV-NC did not affect nuclear or cytoplasmic TFEB distribution. Compared to the negative control, LV-NC and LV-TFEB groups, the AOPP, LV-NC + AOPP and LV-TFEB + AOPP groups exhibited significantly lower nuclear/cytoplasmic ratios of TFEB expression respectively. The total protein expression of TFEB in LV-TFEB cells was also significantly higher than that in LV-NC cells, as assessed by western blot analysis (Fig. 5B). Similarly, we investigated whether overexpression of TFEB affected the AOPP-induced inhibition of autophagy and lysosomal biogenesis in KGN and COV434 cells. Compared with the group treated with AOPPs alone, the LV-TFEB + AOPP group exhibited decreased expression of p62 protein and increased levels of LC3B II protein, whereas no significant differences were observed between the LV-NC + AOPP group and the AOPP group (Fig. 5B). The results of the RFP-GFP-LC3 adenovirus transfection experiment also showed that there were significantly more autophagosomes in the LV-TFEB group than in the control group following AOPP challenge (Fig. 5C). The number of lysosomes detected by Lyso-Tracker and LAMP1 fluorescence was significantly greater in the LV-TFEB + AOPP group but was not greater in the LV-NC + AOPP group than in the AOPP group (Fig. 5D, E). These results suggested that the impairment of autophagic flux and lysosomal biogenesis was significantly alleviated after overexpression of TFEB, promoting TFEB nuclear translocation in granulosa cells. Collectively, our findings clearly confirm that AOPPs inhibit granulosa cell autophagy and lysosome biogenesis through the ROS-mTOR-TFEB signaling pathway may be one of the molecular mechanisms by which AOPP participates in the pathogenesis of POI.

### Chronic AOPP loading in vivo inhibited autophagy and lysosomal biogenesis in the granulosa cells of Sprague-Dawley rats

**Fig. 6:**
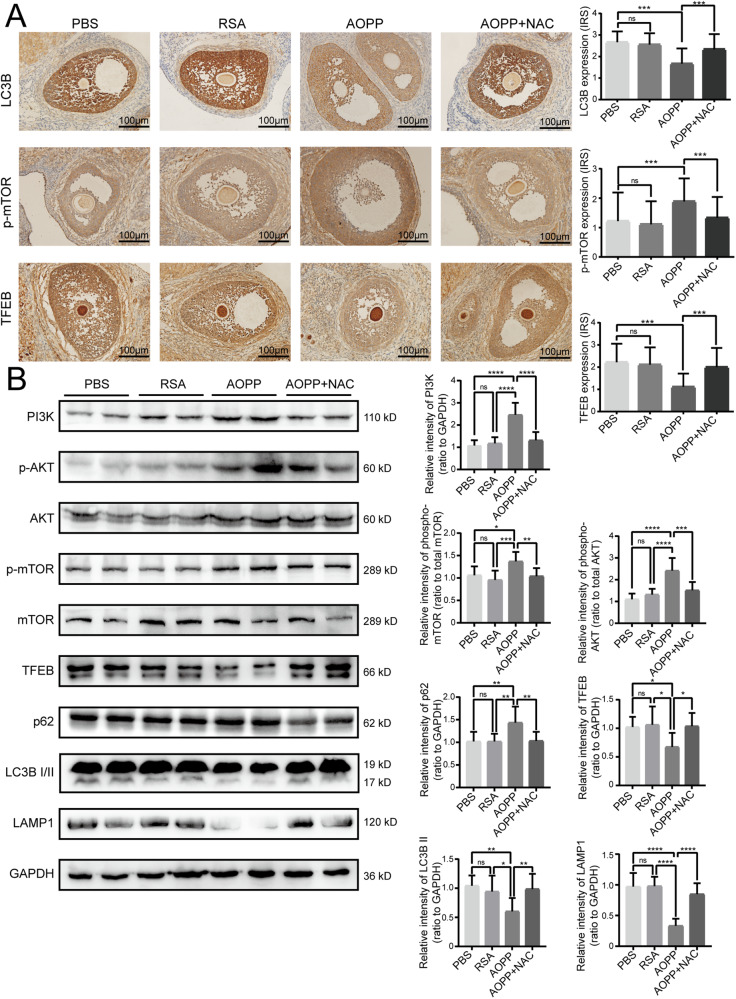
Chronic AOPP loading in vivo inhibited autophagy and lysosomal biogenesis in granulosa cells of Sprague-Dawley rats. **A** Representative images of immunohistochemical staining for LC3, p-mTOR, and TFEB in granulosa cells of rats in the PBS group, RSA group, AOPP group, and AOPP + NAC group (*n* = 9). **B** Representative blots and quantification showing PI3K, AKT, p-AKT, mTOR, p-mTOR, TFEB, p62, LC3B, and LAMP1 in the PBS group, RSA group, AOPP group, and AOPP + NAC group (*n* = 9). The results are expressed as the mean ± SD from experiments. *P < 0.05, **P < 0.01, ***P < 0.001, ****P < 0.0001. Statistical significance was determined by one-way ANOVA and Tukey tests for post hoc comparisons.

To verify the above results in vivo, we treated female Sprague-Dawley rats with AOPPs daily via intraperitoneal injection for 12 weeks and detected the expression of proteins in the mTOR-TFEB pathway and proteins involved in autophagy and lysosomal biogenesis. The immunohistochemistry assay revealed significantly lower LC3B levels, higher p-mTOR levels and lower TFEB levels in granulosa cells in AOPP-treated rats than in PBS-treated rats (Fig. 6A). As shown in Fig. 6B, the western blot results further demonstrated that PI3K, AKT and mTOR phosphorylation was significantly increased and that TFEB levels were significantly decreased in AOPP-treated rats, while NAC treatment reduced these effects. AOPP stimulation in vivo impaired autophagy and lysosomal biogenesis, decreased LC3B II and LAMP1 levels, and increased p62 levels, but NAC administration reversed the AOPP-induced suppression of autophagy and lysosomal biogenesis in rat ovaries.

**Fig. 7:**
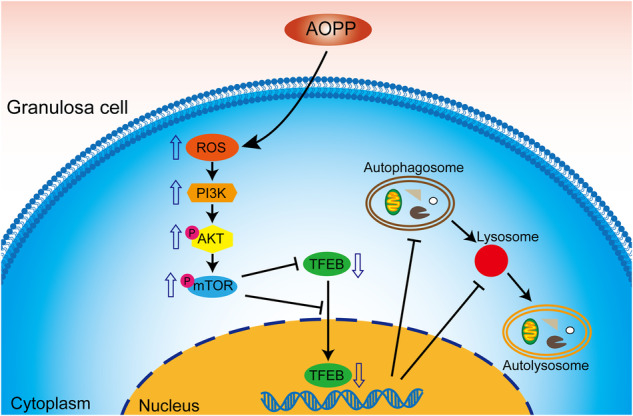
A model of AOPPs attenuating the autophagy-lysosome pathway in ovarian granulosa cells by modulating the ROS-dependent mTOR-TFEB pathway. AOPP-induced accumulation of intracellular ROS activated the PI3K-AKT-mTOR pathway, which resulted in downregulation of TFEB and hindered TFEB translocation to the nucleus, thereby inhibiting autophagy and lysosomal biogenesis in granulosa cells.

Therefore, we confirmed that AOPPs could induce autophagy inhibition and lysosomal biogenesis dysfunction in ovarian granulosa cells via the ROS-dependent mTOR-TFEB signaling pathway both in vitro and in vivo (Fig. 7)_._

## Discussion

The present study provides the first evidence that AOPPs stimulate autophagy inhibition and lysosomal biogenesis dysfunction in ovarian granulosa cells in a process mediated mainly by the ROS-dependent mTOR-TFEB signaling pathway. We found that TFEB function was impaired following AOPP challenge, as reflected by reduced TFEB expression and translocation to the nucleus, and that this was mediated at least in part by ROS-mTOR signaling.

Ovarian deficiency represents a continuum of pathophysiological changes in the ovarian reserve, including a diminished ovarian reserve, premature menopause, and POI, due to accelerated depletion of the primordial follicle pool [22]. The pathogenesis of POI remains largely unclear; thus, no successful treatment for this disorder is available. Oxidative stress induced by various intra- and extracellular stimuli has been confirmed to be involved in ovarian disorders and to display unique functions in disrupting primordial follicle activation and development [23]. AOPPs, as novel markers of oxidative stress, play key roles as inflammatory mediators in multiple chronic diseases [24, 25]. Our previous study revealed that AOPP challenge suppressed cell cycle progression in ovarian granulosa cells through ROS-dependent JNK/p38 MAPK signaling, inhibited granulosa cell proliferation and accelerated follicle atresia in rat models [8]. The present study provides a novel perspective on AOPP-mediated dysfunction of autophagy and lysosomal biogenesis, further confirming the vital roles of AOPPs in the pathogenesis of POI.

Autophagy displays unique functions in maintaining oocytes and in primordial follicle development and atresia [26]. In recent years, the relationship between autophagy and POI has been further investigated. Impaired autophagic flux has been observed in several experimental models of POI, and several genetic alterations causing autophagy defects have been found to exacerbate the development of POI in mice. For instance, germ cell-specific knockout of Atg7 in mice results in autophagy deficiency and severe excessive ovarian follicle loss, which is very similar to human POI progression, mainly by drastically reducing the number of primordial follicles at the neonatal transition period [27]. Mutations in ATG7 (p.Arg403Leu) and ATG9A (p.Arg758Cys) have also been described to be involved in the pathogenesis of human POI, resulting in a dramatic decline in autophagosome biogenesis and consequent impairment of autophagic flux, which is a crucial biological process implicated in the preservation of primordial follicles that form the ovarian reserve [10]. Shao et al. reported that the insufficient autophagic pathway in granulosa cells of biochemical POI induced the accumulation of WT1 protein, thus impairing granulosa cell differentiation [12]. Furthermore, autophagy defects impair homologous recombination repair ability [28], reducing the proliferation capacity of granulosa cells after DNA damage and thus participating in the occurrence of POI by affecting granulosa cell differentiation and DNA damage repair [29]. Previous studies have demonstrated that AOPPs can inhibit the biogenesis of autophagosomes in renal tubular epithelial cells [13]. However, Liao et al. found that AOPPs mainly decrease the activity of lysosomal proteolytic enzymes, blocking autophagic flux in macrophages [14]. The results of our study suggest that elevated AOPP levels in patients with POI inhibit granulosa cell autophagy and lysosomal biogenesis may be a significant mechanism by which AOPPs participate in the pathogenesis of POI.

TFEB is a transcription factor containing basic helix-loop-helix and leucine zipper domains and is characterized as a master regulator of autophagy and lysosomal biogenesis [30]. TFEB has been well demonstrated to play pivotal roles in various human diseases and several biological processes, including autophagy, apoptosis, inflammation and survival [31–33]. Since TFEB was recently discovered to be a potential therapeutic target for alleviation of myocardial ischemic injury and neurodegeneration [34, 35], we were interested in its role in regulating the autophagy-lysosome pathway in ovarian diseases. In our research, AOPP challenge decreased TFEB expression and suppressed TFEB nuclear translocation, which contributed to autophagy inhibition and lysosomal biogenesis impairment. Moreover, lentivirus-mediated overexpression of TFEB mitigated AOPP-induced autophagy inhibition and lysosomal biogenesis impairment in KGN cells. These findings indicated the potential value of TFEB as a therapeutic target in diseases featuring autophagy dysfunction (including POI). However, the specific role of TFEB in the pathogenesis of POI warrants further investigation.

mTOR inactivation-induced TFEB dephosphorylation has been proposed to lead to TFEB translocation to the nucleus, which activates the transcription of specific targeted genes [36]. On the one hand, the mTOR signaling cascade is involved in ovarian follicle development, in which it regulates granulosa cell proliferation, differentiation and ovulation [37]. However, inhibition of mTOR signaling in primordial follicle oocytes does not affect follicle development or female fertility, suggesting that activation of mTOR signaling in granulosa cells is a priming signal for primordial follicle activation [38]. The mTOR pathway also exerts a regulatory effect on natural ovulation, and inhibition of the mTOR signaling pathway causes preovulatory follicle maturation, resulting in anovulation, which is a key characteristic of POI [39]. On the other hand, the mTOR pathway also acts as a transmitter of exogenous stimuli involved in the development of follicles. Aberrant activation of mTOR signaling accelerates granulosa cell differentiation and increases primordial follicle activation, ultimately leading to accelerated ovarian reserve decline. For instance, abnormal accumulation of bisphenol A, an endocrine-disrupting chemical, might influence human ovarian functions, leading to abnormal folliculogenesis by activating autophagy in granulosa cells through an mTOR-mediated pathway [40]. Our present results suggested that AOPPs inhibited autophagic flux and lysosomal biogenesis in ovarian granulosa cells by activating the mTOR pathway. Rapamycin, a specific mTOR inhibitor, attenuated the AOPP-induced decreases in TFEB protein levels and impairment of nuclear translocation due in part to AOPP-induced mTOR activation in KGN cells, further supporting a critical role for mTOR in regulating AOPP-induced inactivation of TFEB. It is probable that a more potent mTOR inhibitor (if available) may be able to ameliorate TFEB dysfunction in the ovaries and in turn offer enhanced protection against AOPP-induced autophagy inhibition.

ROS accumulation is a key process in the pathogenesis of AOPP. We found that AOPPs also increased intracellular ROS levels in granulosa cells, thereby inducing autophagy inhibition and lysosome biogenesis dysfunction, and that this effect was alleviated by pretreatment with the ROS scavenger NAC. Furthermore, NAC abolished AOPP-induced TFEB cytoplasmic retention and autophagy inhibition, implying that ROS were the primary upstream factors responsible for these effects during AOPP challenge. The cumulative lines of evidence suggest that ROS and autophagy regulation play bidirectional roles, and multiple studies have shown that exogenous stress leads to increases in intracellular ROS levels, inhibition of the mTOR signaling pathway, and an increased level of autophagy [41]. In the ovaries, chemotherapeutic drugs and environmental endocrine disrupters can cause dramatic increases in ROS in granulosa cells and activation of autophagy pathways, leading to apoptosis of granulosa cells and follicle atresia, which account for the occurrence of POI [42, 43]. However, the cellular autophagy level may be suppressed when intracellular ROS levels are only mildly increased [44, 45]. Therefore, we speculate that the AOPP-induced intracellular ROS elevation was not drastic enough to reach the threshold of ROS-induced autophagy activation. Under chronic AOPP challenge, the activation of autophagy may be inhibited, and lysosomal biogenesis may be blocked.

We found that AOPP-induced accumulation of intracellular ROS activated the mTOR pathway, which resulted in downregulation of TFEB and hindered TFEB translocation to the nucleus, thereby inhibiting autophagy and lysosomal biogenesis in KGN cells. Our findings indicate that removing intracellular ROS, inhibiting mTOR or overexpressing TFEB may protect against AOPP-induced autophagy inhibition and lysosomal biogenesis. The possible cellular events that lead to impairment of autophagic flux and lysosomal biogenesis via ROS-mTOR-TFEB signaling and granulosa cell autophagy dysfunction may be associated with a molecular mechanism by which AOPPs participate in the pathogenesis of POI. Thus, targeting AOPP-induced autophagy and lysosomal biogenesis disorders might be a novel therapeutic strategy for early prevention and treatment of POI.

## Materials and methods

### AOPP preparation

AOPPs were prepared in vitro by incubation with BSA or RSA (20 mg/mL; Sigma, St. Louis, MO, USA) with 40 mM HOCl (Sigma) for 30 min according to a procedure described previously [8]. The prepared samples were dialyzed against 100 times the volume of PBS for 24 h at 4 °C, during which the solution was changed every 6 h to remove free HOCl completely. All samples were passed through a Detoxi-Gel column (Thermo Scientific, Waltham, MA, USA) to remove endotoxin. The endotoxin levels in samples were below 0.05 ng/mg protein which were measured by a Limulus Amoebocyte Lysate kit (BioWhittaker, Walkersville, MD, USA).

### Cell culture and lentivirus transfection

The immortalized human ovarian granulosa-like tumor cells (KGN and COV434), obtained from the Procell Life Science & Technology Co.,Ltd. (Wuhan, China), were used for in vitro functional and mechanistic studies. KGN and COV434 cells were cultured in Dulbecco’s modified Eagle medium (DMEM)/F-12 (Gibco, Carlsbad, CA, USA) supplemented with 10% fetal bovine serum (Gibco) and maintained at 37 °C in a 5% CO_2_ atmosphere. A lentivirus for TFEB overexpression (LV-TFEB) and the empty vector (LV-normal control; LV-NC) were obtained from GeneChem (Shanghai, China). LV-TFEB and LV-NC were transfected into KGN and COV434 cells according to the manufacturer’s manual and then treated with AOPPs before further analysis. Western blotting was used to examine the efficiency of TFEB overexpression.

### Western blot analysis and isolation of nuclear and cytoplasmic proteins

Cultured KGN/COV434 cells or rat ovary samples were lysed on ice in radioimmunoprecipitation assay (RIPA) buffer with phenylmethanesulfonyl fluoride (PMSF), protease inhibitor cocktail, and phosphatase inhibitor (Beyotime, Shanghai, China). After centrifugation at 13,000 rpm and 4 °C for 15 min, the protein concentration was determined using a Bicinchoninic Acid (BCA) Protein Assay Kit (Beyotime). The proteins (30 μg) were separated by SDS-polyacrylamide gel electrophoresis (PAGE) using 8–12% acrylamide gels and then transferred to polyvinylidene fluoride (PVDF) membranes (Bio-Rad Laboratories, Hercules, CA, USA). After incubation with 5% BSA (Sigma) for 1 h at room temperature (RT), the PVDF membranes were incubated overnight at 4 °C with specific primary antibodies. Following washing with TBST 3 times, the membranes were incubated with horseradish peroxidase (HRP)-conjugated secondary antibodies (Cell Signaling Technology [CST], Beverly, MA, USA) for 1 h at RT. After washing with TBST 3 times again, the protein bands were visualized with Clarity Western Enhanced Chemiluminescence (ECL) Substrate (Bio-Rad Laboratories). ImageJ software was used to analyze and normalize the band intensities, and each band intensity was normalized to that of glyceraldehyde-3-phosphate dehydrogenase (GAPDH). The following primary antibodies were used: anti-mouse SQSTM1/p62 (ab56416, Abcam, Cambridge, UK), anti-rabbit LC3B (A5202, Bimake, Houston, TX, USA), anti-mouse GAPDH (60004-1-Ig, GAPDH, Proteintech, Wuhan, China), anti-rabbit p-mTOR (ab109268, Abcam), anti-rabbit mTOR (ab134903, Abcam), anti-rabbit AKT (4691, CST), anti-rabbit p-AKT (4060, CST), anti-rabbit PI3K (ab191606, Abcam), and anti-rabbit TFEB (ab170604, Abcam). The nuclear and cytoplasmic protein were separated using the Nuclear and Cytoplasmic Protein Extraction kit according to the manufacturer’s instructions (Beyotime).

### Transmission electron microscopy

KGN and COV434 cells were fixed with 2.5% glutaraldehyde at RT for 30 min and then at 4 °C for 24 h. After three washes with PBS, the KGN and COV434 cells were fixed with 1% osmium at RT for 2 h, dehydrated via sequential incubations in an increasing gradient of ethanol, and incubated in 100% acetone. The samples were permeated with an SPI-Pon 812 Embedding Kit (SPI, West Chester, PA, USA). The sliced sections were double-stained with uranium lead and dried overnight at RT, and autolysosomes and autophagosomes were observed by transmission electron microscopy (HT7700; Hitachi, Tokyo, Japan).

### Tandem RFP- GFP-LC3 assay

To investigate autophagic flux, KGN cells were transfected with an RFP-GFP-LC3 adenovirus according to the manufacturer’s protocols. In brief, the RFP-GFP-LC3 adenovirus (GeneChem) was transfected into the cells, and the transfection solution was replaced with complete medium after 8 h. After 48 h, the cells were fixed with 4% paraformaldehyde for 20 min and washed three times with PBS. The nuclei were stained with DAPI (blue) for 15 min in the dark. The samples were observed and photographed with a Zeiss LSM 980 confocal microscope (Zeiss, Oberkochen, Germany). The RFP-GFP-LC3 protein initially labeled the membranes of autophagosomes and was then delivered to autolysosomes. The GFP fluorescent signal was sensitive to and quenched by the acidic conditions within lysosomes, while the RFP fluorescent signal was stable in lysosomes. The yellow puncta with GFP and RFP signals indicated autophagosomes that had not yet fused with lysosomes, and the red puncta without GFP signals indicated autolysosomes. Quantification of the GFP- and RFP-positive puncta and colocalization between the two different signals were examined.

### Immunofluorescence staining

KGN and COV434 cells were grown on confocal dishes for 48 h and treated with 200 μg/mL AOPP. After 3 washes with PBS, the cells were fixed in 4% paraformaldehyde at RT for 20 min, permeabilized in 0.1% Triton-X 100 for 15 min, and then blocked in 5% BSA at 37 °C for 30 min. The cells were then incubated with the anti-rabbit TFEB (ab170604, Abcam) and LAMP1 (ab62562, Abcam) at 4 °C overnight, further incubated with CoraLite488-conjugated anti-rabbit antibodies (SA00013-2, Proteintech) or CoraLite594-conjugated anti-rabbit antibodies (SA00013-4, Proteintech) at 37 °C for 1 h and finally incubated with DAPI for 5 min. Images were acquired with a LSM 980 confocal microscope (Zeiss), and image analysis was performed with ImageJ software.

### Lyso-Tracker assay

Lyso-Tracker was used to assess the numbers of lysosomes in living cells following the manufacturer’s instructions. KGN and COV434 cells were seeded on confocal dishes for 24 h for attachment and then cultured for another 48 h. Then, the cells were incubated in the dark with 50 nM Lyso-Tracker Red (Beyotime) at 37 °C for 30 min. Finally, the cells were incubated with Hoechst 33342 Staining Solution for Live Cells (Beyotime) in growth medium for 10 min to achieve nuclear DNA staining. The fluorescence intensity was observed under a LSM 980 confocal microscope (Zeiss), and image analysis was performed with ImageJ software.

### Determination of Intracellular ROS Generation

2′,7′-dichlorofluorescein diacetate (DCFH-DA) (Sigma) was used to detect intracellular ROS. KGN cells and COV434 cells were treated with AOPP or NAC + AOPP, and then incubated with 10 μM DCFH-DA for 30 min at 37 °C. Fluorescence intensity (Ex/Em = 488/525) was measured on a fluorescence microplate reader (Spectra Max M5, Molecular Devices, Sunnyvale, CA, USA).

### Animal procedures

The animal experiments are described in detail in our latest publication [8]. Briefly, 36 female Sprague-Dawley rats (7 weeks old, weighing 200-230 g) were randomized into four groups (*n* = 9 per group) with daily intraperitoneal injection of PBS, unmodified RSA (50 mg/kg), AOPPs (50 mg/kg) or AOPPs (50 mg/kg) + N-acetylcysteine (NAC) (200 mg/kg) for 12 weeks. An online sample size calculator (https://clincalc.com/stats/samplesize.aspx) was used for calculations for sample sizes. The allocation of mice in each group were randomized in accordance with the random number table and blinded. The ovaries were isolated immediately after the rats were sacrificed, and the right ovaries were fixed in 4% formaldehyde for immunohistochemical analysis. The left ovaries were placed into liquid nitrogen and then stored at −80 °C for subsequent western blot analysis. All animal studies were performed in accordance with guidelines set by the Nanfang hospital animal ethic committee.

### Immunohistochemistry

Embedded ovary samples were sliced into 4 μm sections, deparaffinized in xylene, rehydrated in a graded series of alcohols, and subjected to antigen retrieval with 3% hydrogen peroxide. Then, the sections were blocked in goat serum and subsequently incubated with the corresponding primary antibodies. The ovary slices were incubated with a secondary antibody labeled with biotin and stained with diaminobenzidine (DAB, Zhongshanjinqiao, Beijing, China). Images were scanned under an Olympus BX63 microscope (Olympus, Tokyo, Japan).

### Statistical analysis

All experimental results are presented as the means ± standard deviations (SDs). Statistical analyses were conducted with SPSS 20.0 (IBM Corp, Armonk, NY, USA) and GraphPad Prism 9.0 (GraphPad Software, San Diego, CA, USA) software. The Kolmogorov–Smirnov test and Levene’s test were used successively to test the normal distribution and homogeneity of variances. Differences in variables between two groups were analyzed using Student’s *t* test, and multiple comparisons were conducted with one-way analysis of variance (ANOVA) followed by the Tukey tests for post- hoc comparisons. All experimental procedures were repeated at least three independent times. A two-sided *P* value of <0.05 was considered to indicate statistical significance.

### Reporting summary

Further information on research design is available in the Nature Research Reporting Summary linked to this article.

### Supplementary information

Original Data File

Reporting Summary

## Data Availability

The data that support the findings of this study are available from the corresponding author upon reasonable request.
