# Supplementary material for: Advanced oxidation protein products attenuate the autophagy-lysosome pathway in ovarian granulosa cells by modulating the ROS-dependent mTOR-TFEB pathway
Source: Cell Death Dis. 2024 Feb 21;15(2):161. doi: 10.1038/s41419-024-06540-w (PMC10881514; doi:10.1038/s41419-024-06540-w)
Supplement: Supplementary file 1 — Original Data File [file 41419_2024_6540_MOESM1_ESM.pdf]

Fig 1A

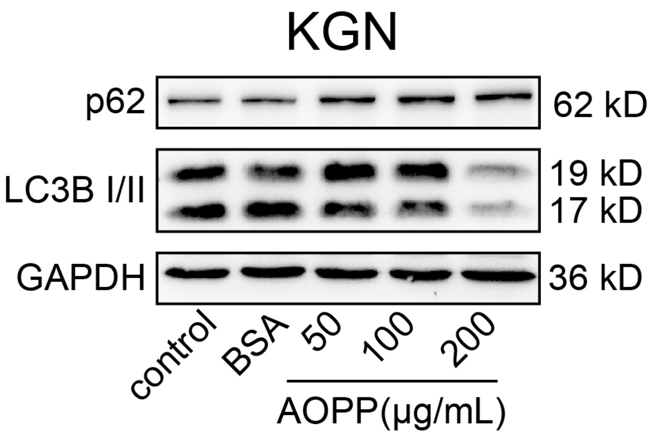

KGN
p62
62 kD
LC3B I/II
19 kD
17 kD
GAPDH
36 kD
control
BSA
50
100
200
AOPP(μg/mL)

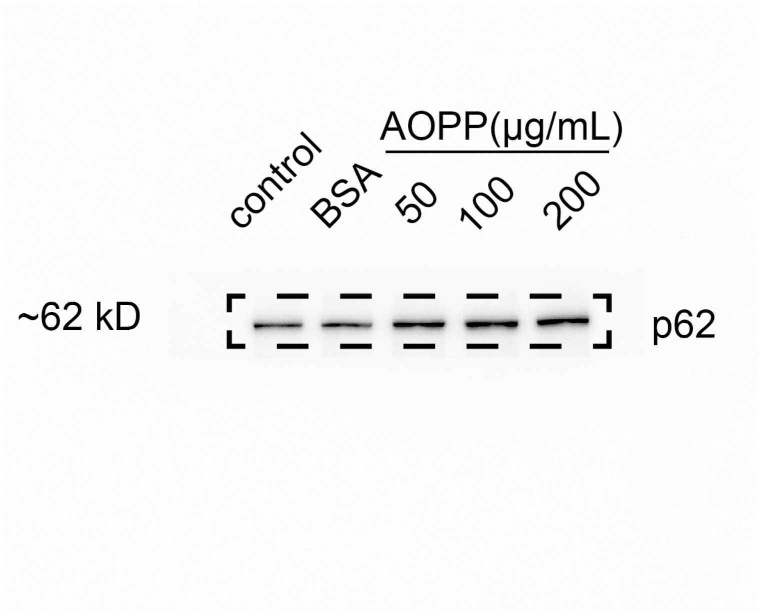

AOPP(μg/mL)
control
BSA
50
100
200
~62 kD
p62

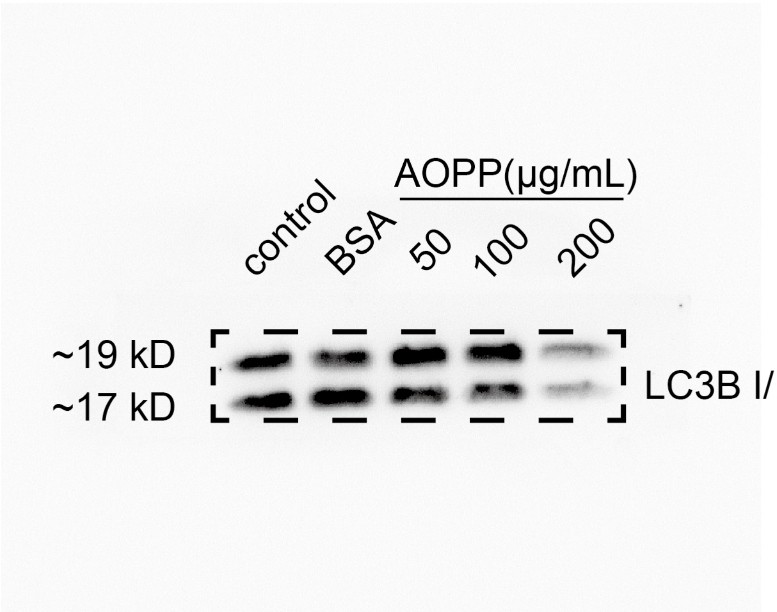

AOPP(μg/mL)
control
BSA
50
100
200
~19 kD
~17 kD
LC3B I/II

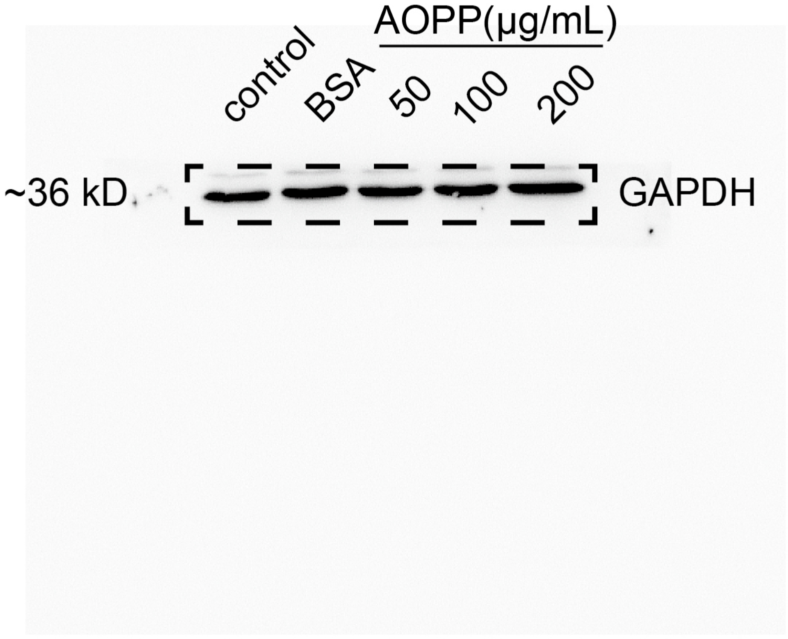

AOPP(μg/mL)
control
BSA
50
100
200
~36 kD
GAPDH

Fig 1A

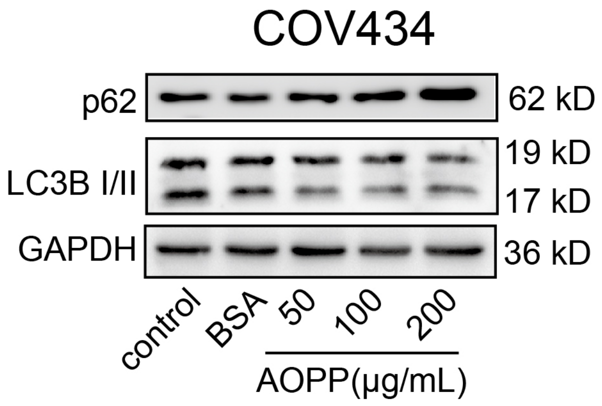

COV434
p62
62 kD
LC3B I/II
19 kD
17 kD
GAPDH
36 kD
control
BSA
50
100
200
AOPP(μg/mL)

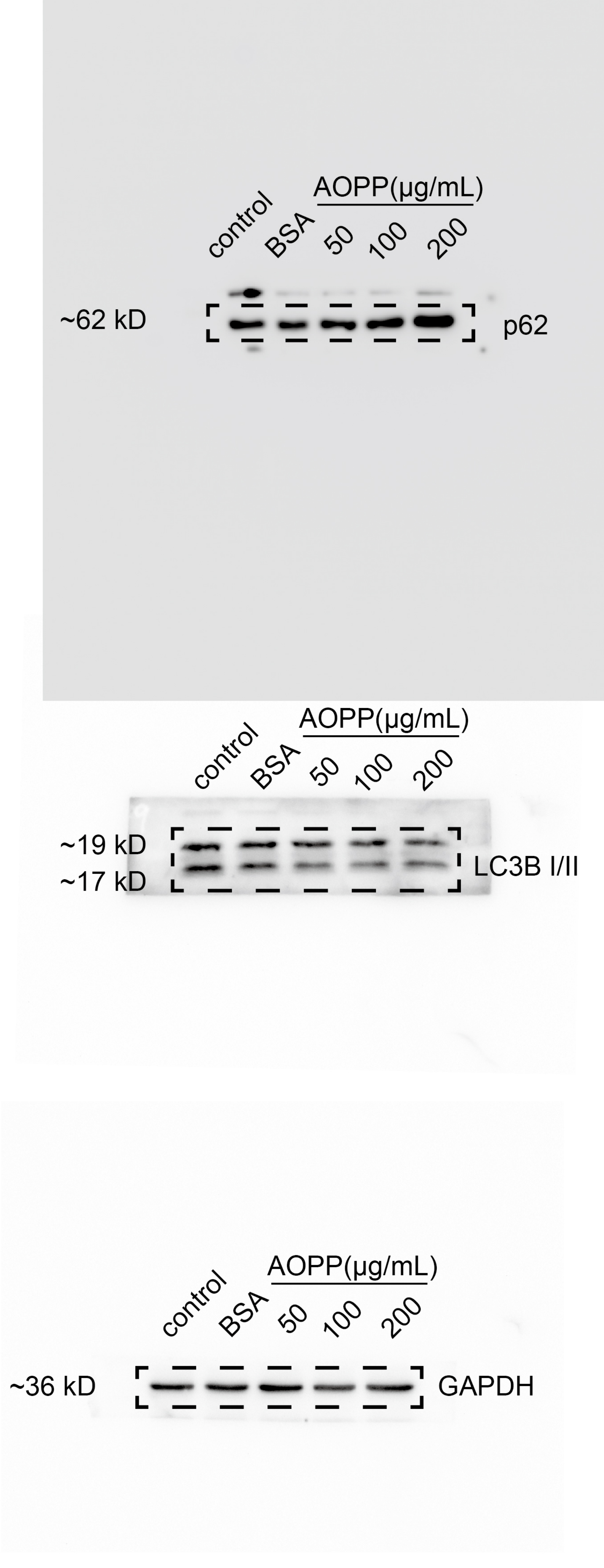

AOPP(μg/mL)
control
BSA
50
100
200
~62 kD
p62
AOPP(μg/mL)
control
BSA
50
100
200
~19 kD
~17 kD
LC3B I/II
AOPP(μg/mL)
control
BSA
50
100
200
~36 kD
GAPDH

Fig 1B

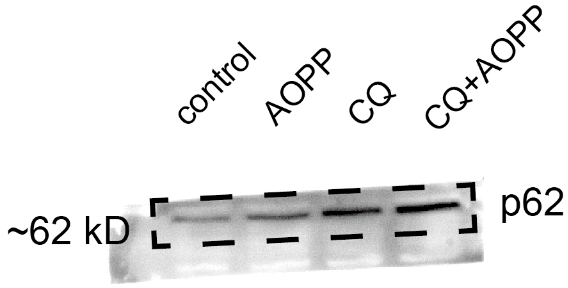
control
AOPP
CQ
CQ+AOPP
~62 kD
p62

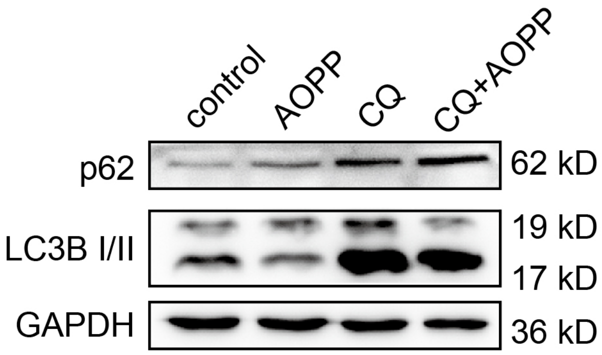
control
AOPP
CQ
CQ+AOPP
p62
62 kD
LC3B I/II
19 kD
17 kD
GAPDH
36 kD

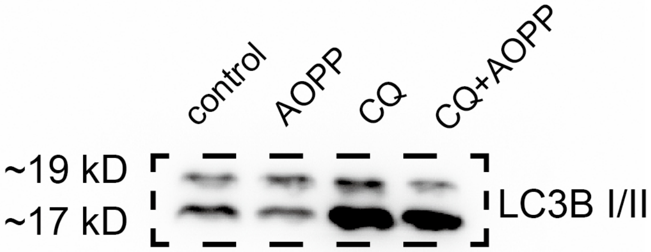
control
AOPP
CQ
CQ+AOPP
~19 kD
~17 kD
LC3B I/II

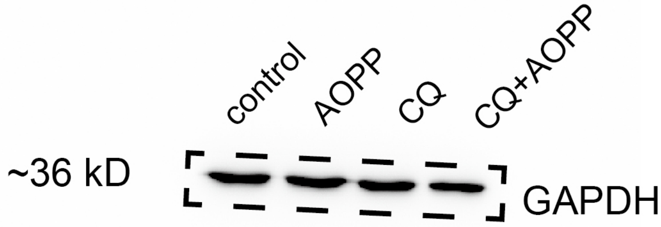
control
AOPP
CQ
CQ+AOPP
~36 kD
GAPDH

Fig 1B

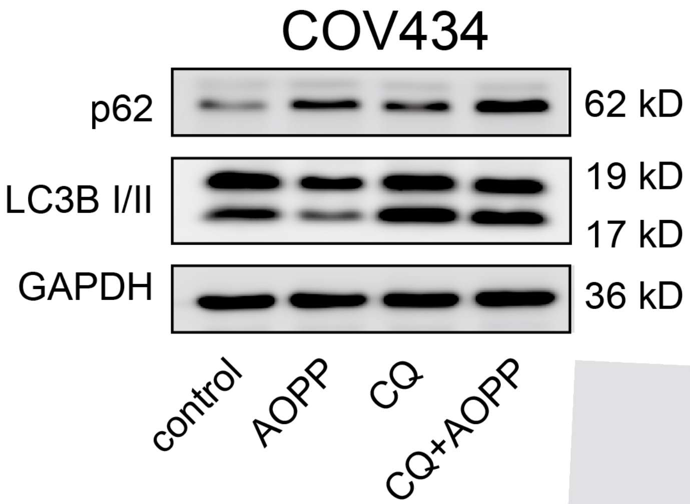
COV434
p62
62 kD
LC3B I/II
19 kD
17 kD
GAPDH
36 kD
control
AOPP
CQ
CQ+AOPP

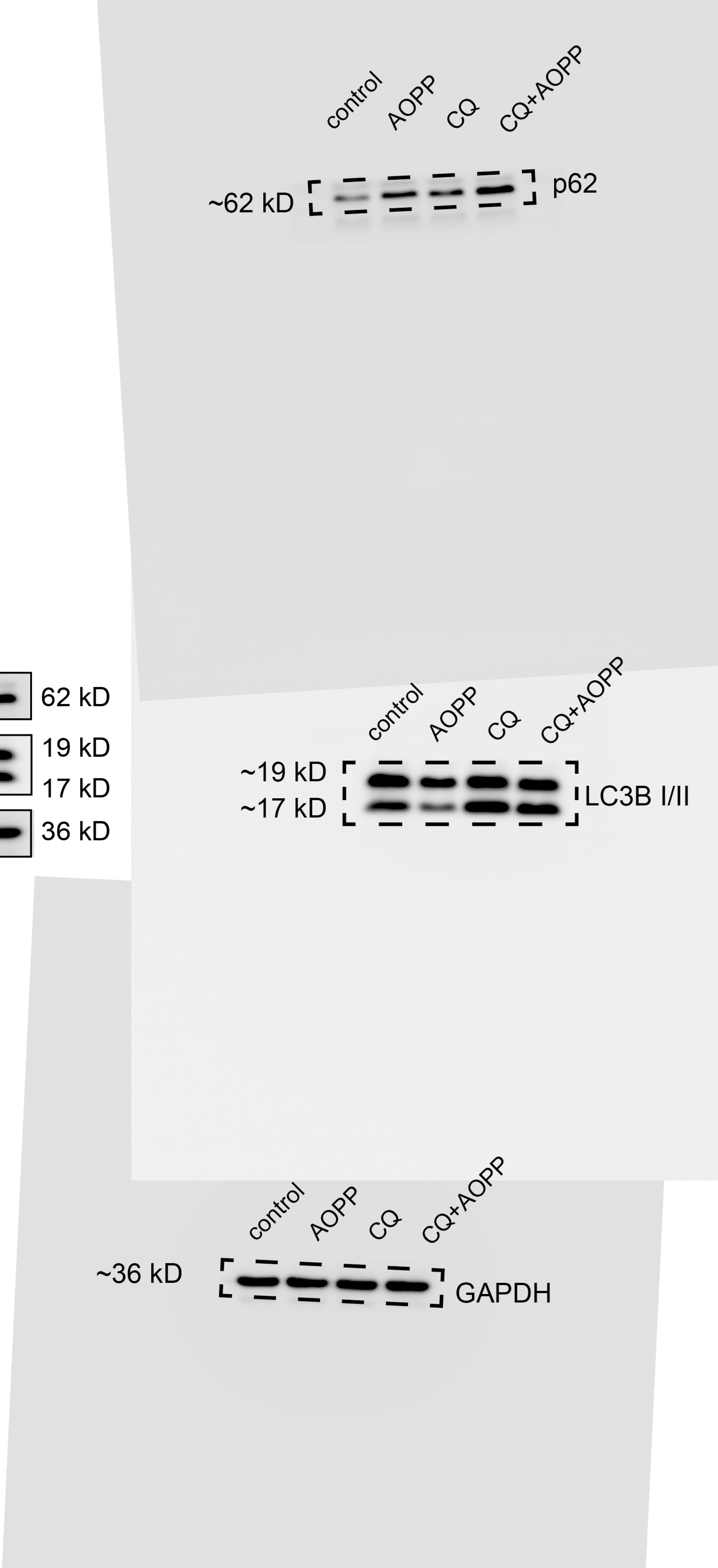
control
AOPP
CQ
CQ+AOPP
~62 kD
p62
control
AOPP
CQ
CQ+AOPP
~19 kD
~17 kD
LC3B I/II
control
AOPP
CQ
CQ+AOPP
~36 kD
GAPDH

Fig 1C

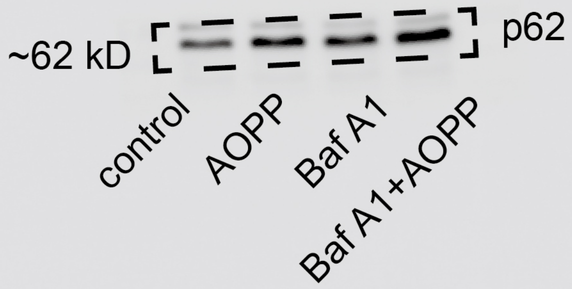

~62 kD
p62
control
AOPP
Baf A1
Baf A1+AOPP

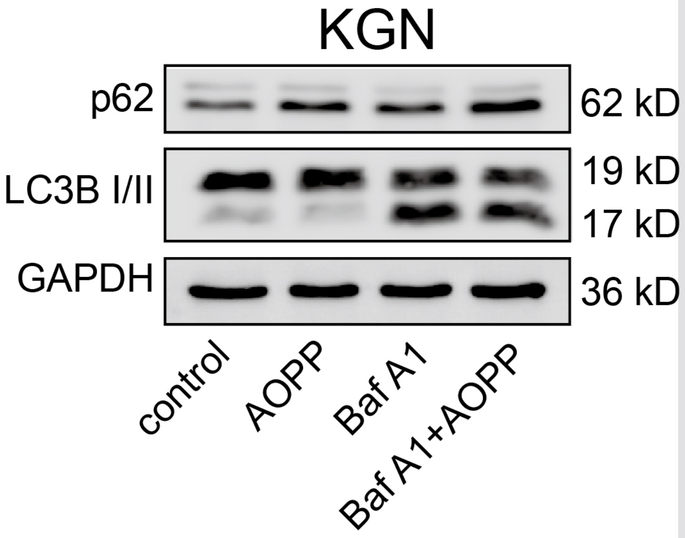

KGN
p62
62 kD
LC3B I/II
19 kD
17 kD
GAPDH
36 kD
control
AOPP
Baf A1
Baf A1+AOPP

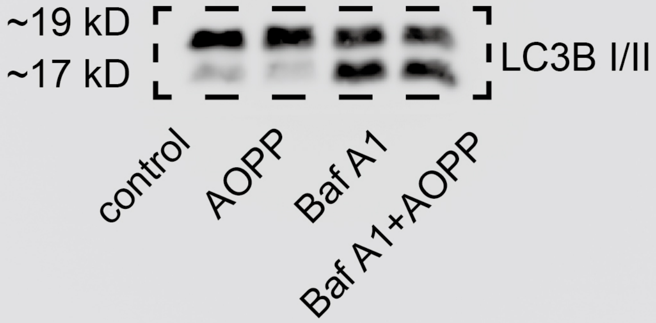

~19 kD
~17 kD
LC3B I/II
control
AOPP
Baf A1
Baf A1+AOPP

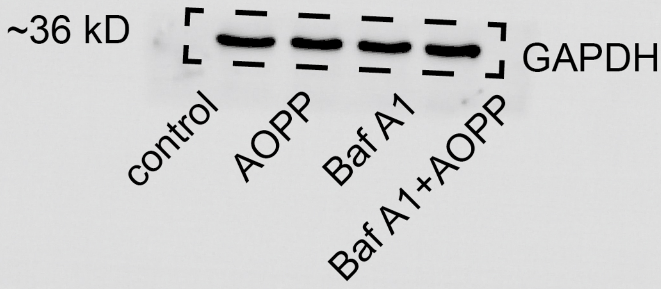

~36 kD
GAPDH
control
AOPP
Baf A1
Baf A1+AOPP

Fig 1C

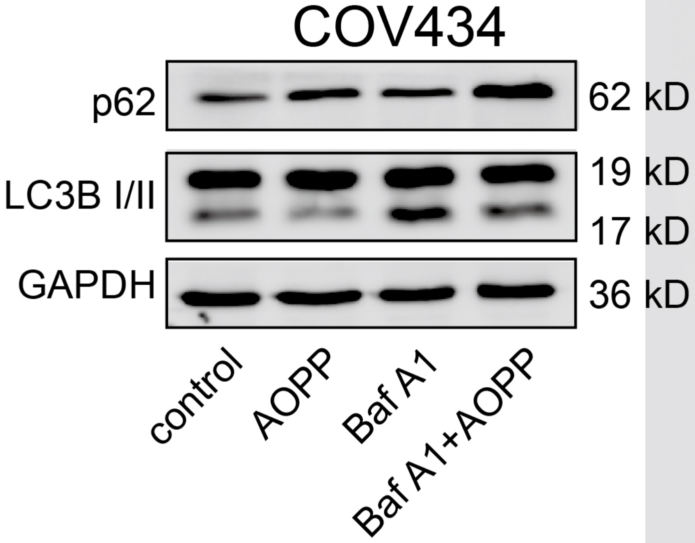
COV434
p62
62 kD
LC3B I/II
19 kD
17 kD
GAPDH
36 kD
control
AOPP
Baf A1
Baf A1+AOPP

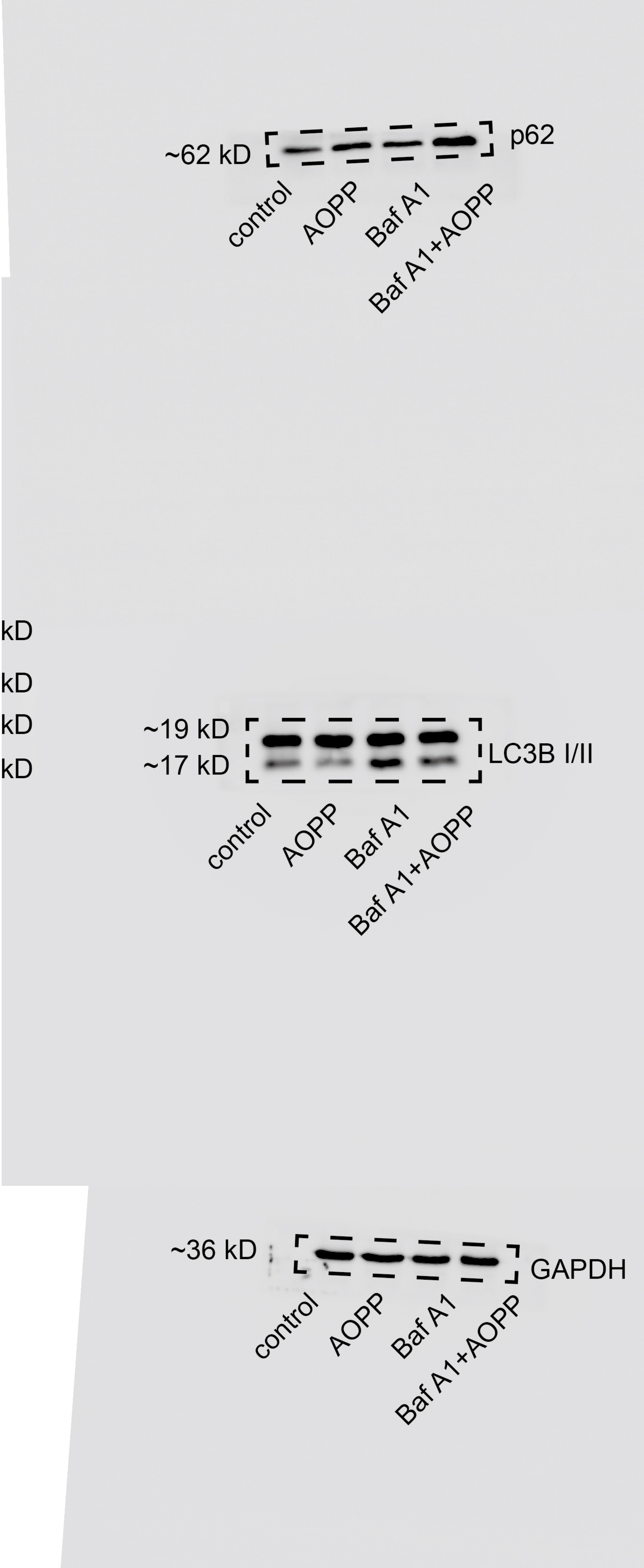
~62 kD
p62
control
AOPP
Baf A1
Baf A1+AOPP
~19 kD
~17 kD
LC3B I/II
control
AOPP
Baf A1
Baf A1+AOPP
~36 kD
GAPDH
control
AOPP
Baf A1
Baf A1+AOPP

Fig 2A

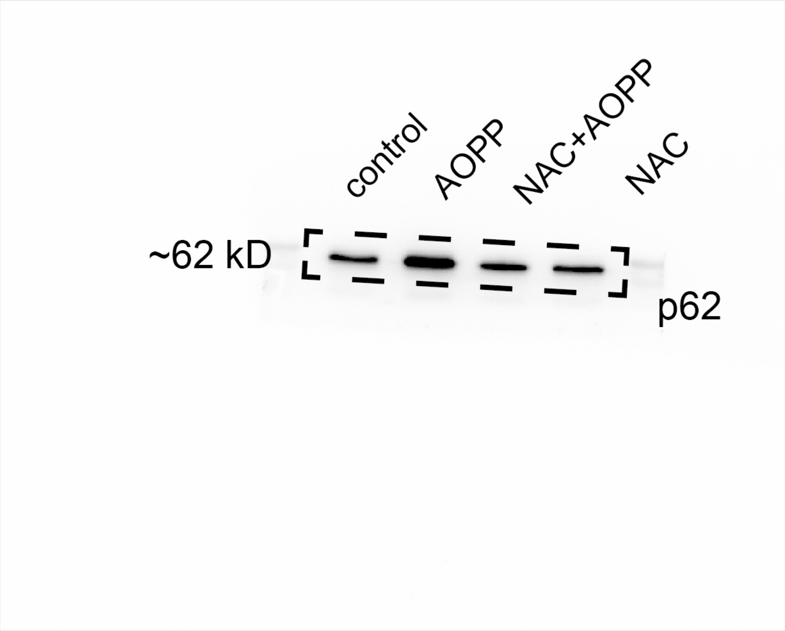
control
AOPP
NAC+AOPP
NAC
~62 kD
p62

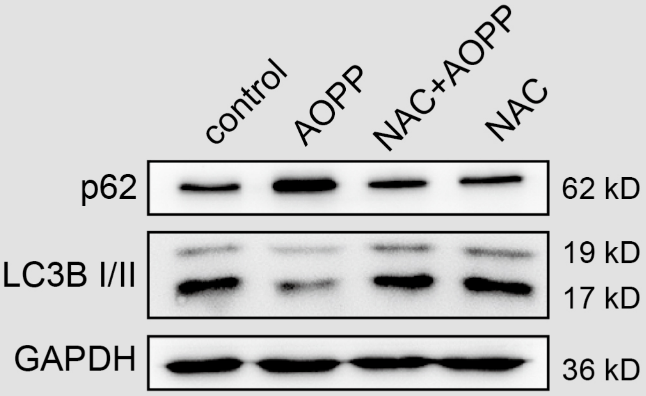
control
AOPP
NAC+AOPP
NAC
p62
62 kD
LC3B I/II
19 kD
17 kD
GAPDH
36 kD

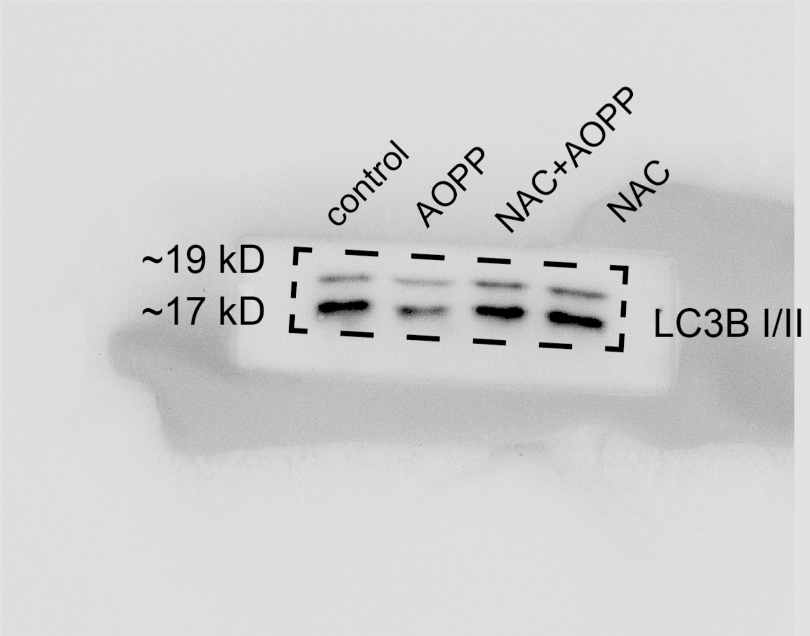
control
AOPP
NAC+AOPP
NAC
~19 kD
~17 kD
LC3B I/II

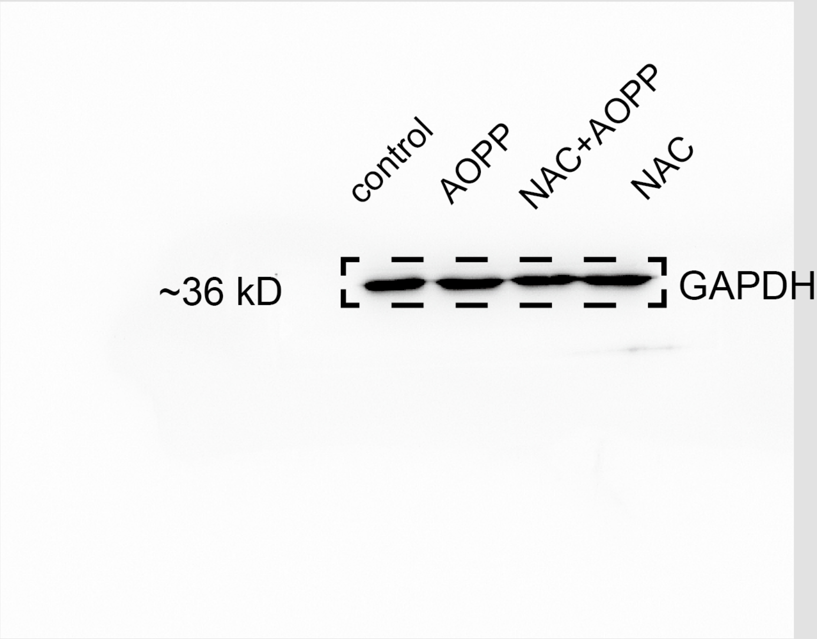
control
AOPP
NAC+AOPP
NAC
~36 kD
GAPDH

Fig 2A

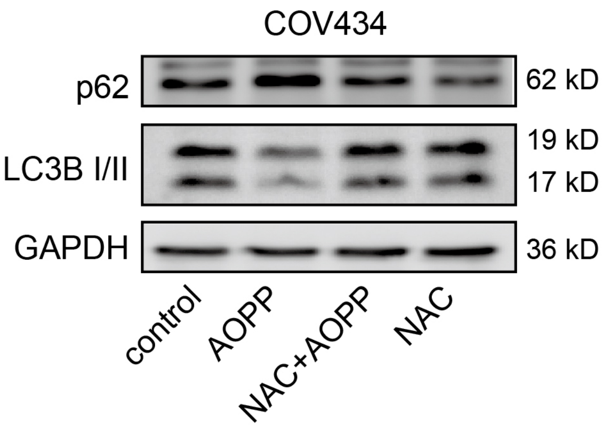
COV434
p62
62 kD
LC3B I/II
19 kD
17 kD
GAPDH
36 kD
control
AOPP
NAC+AOPP
NAC

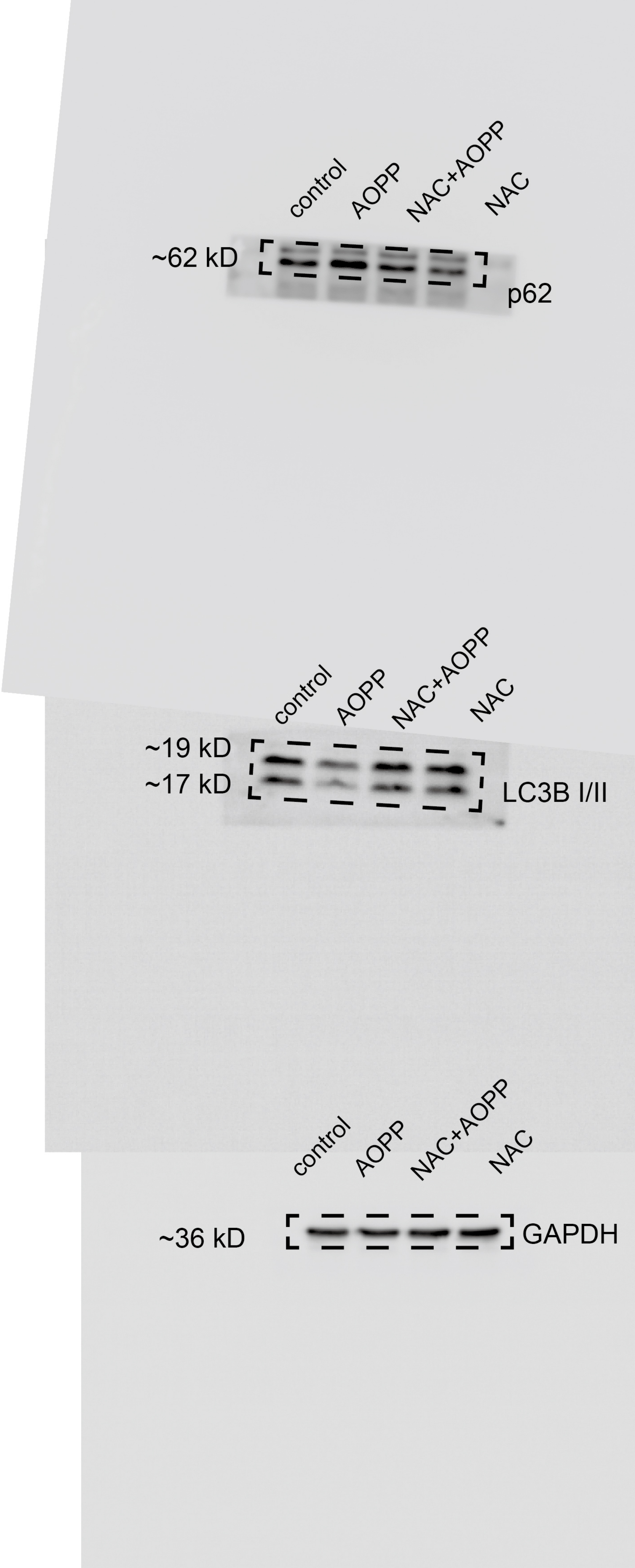
control
AOPP
NAC+AOPP
NAC
~62 kD
p62
control
AOPP
NAC+AOPP
NAC
~19 kD
~17 kD
LC3B I/II
control
AOPP
NAC+AOPP
NAC
~36 kD
GAPDH

Fig 3A

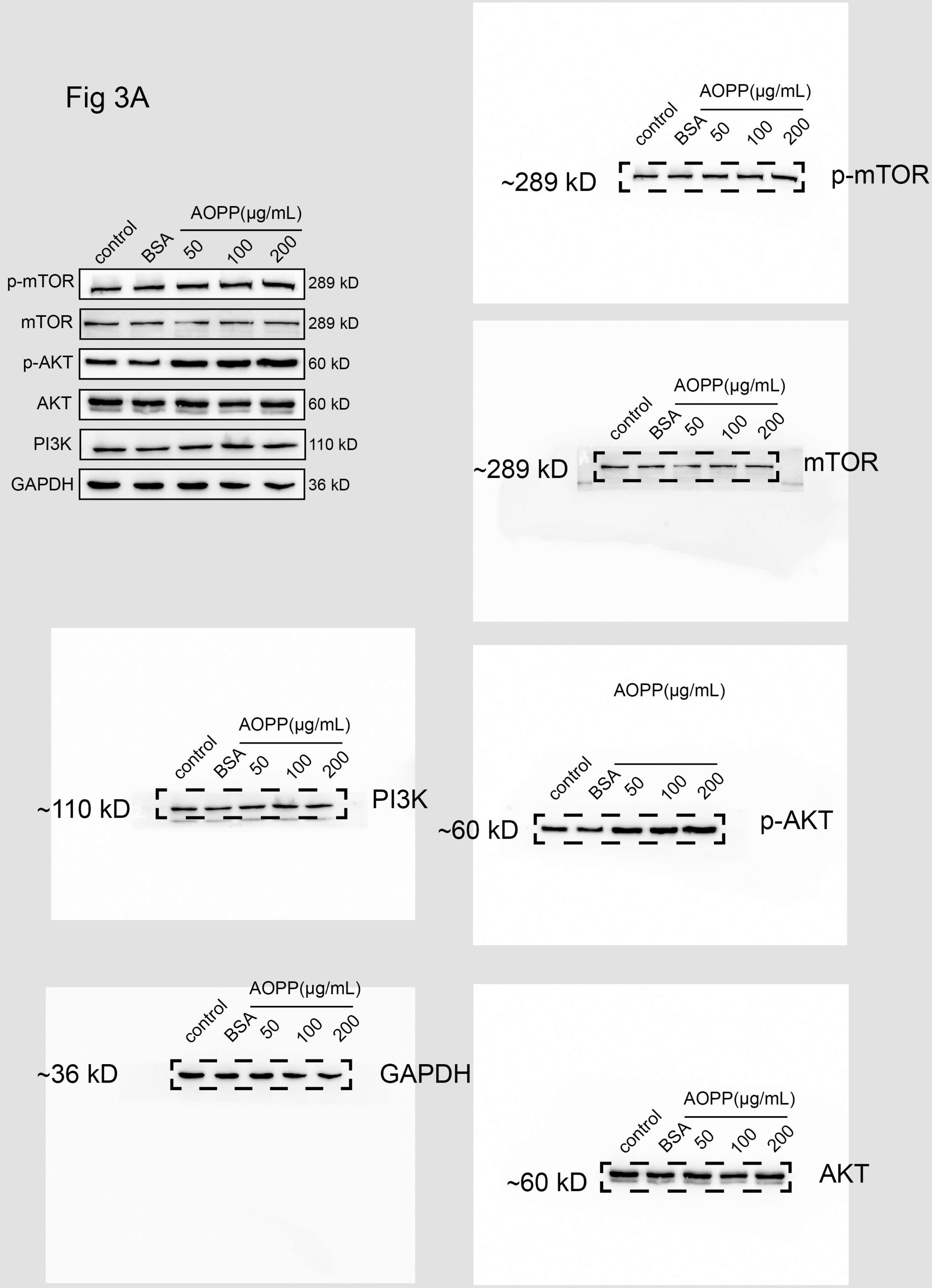

AOPP(μg/mL)
control
BSA
50
100
200
p-mTOR
mTOR
p-AKT
AKT
PI3K
GAPDH
289 kD
289 kD
60 kD
60 kD
110 kD
36 kD
~289 kD
p-mTOR
~289 kD
mTOR
~110 kD
PI3K
~60 kD
p-AKT
~36 kD
GAPDH
~60 kD
AKT

Fig 3A

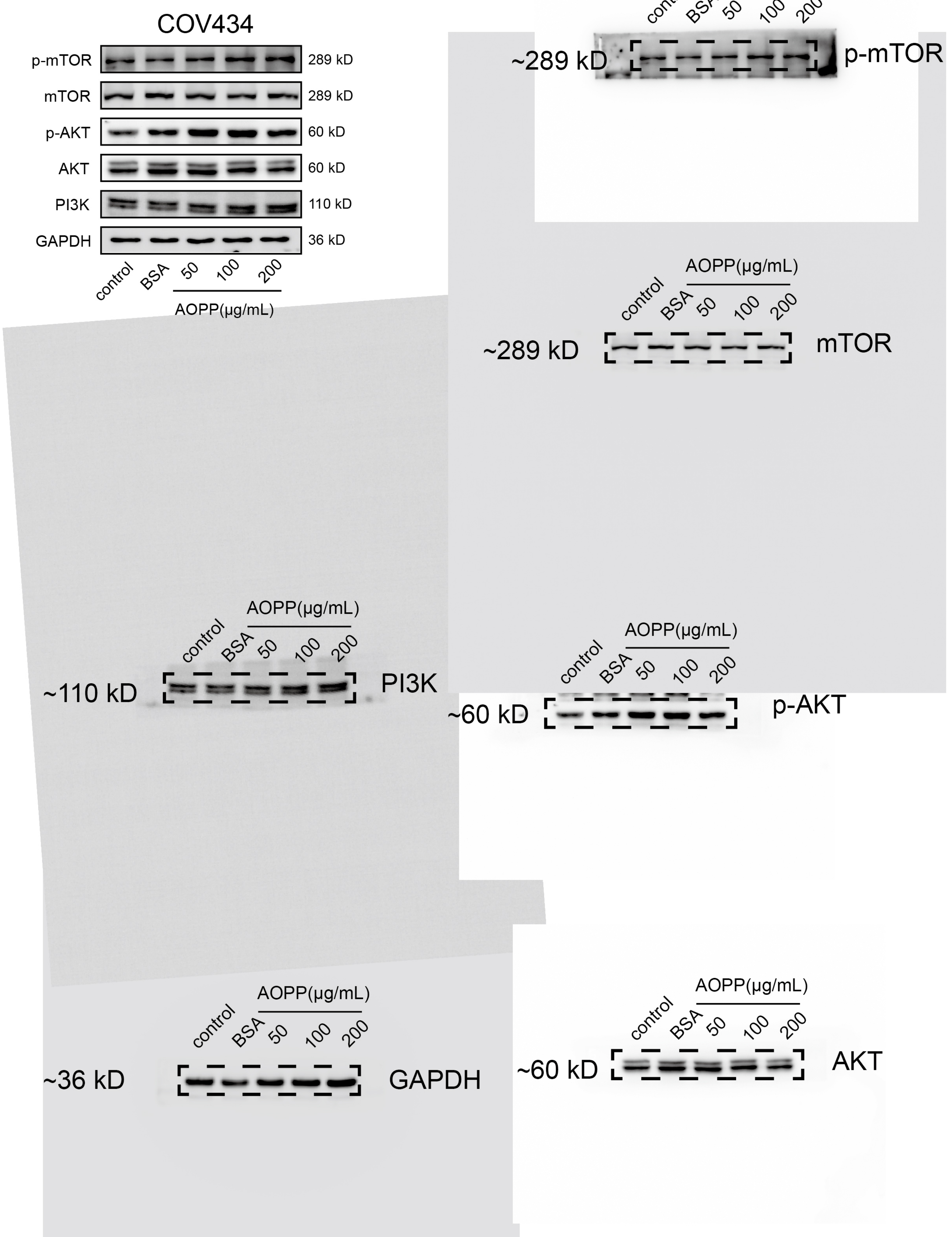
COV434
p-mTOR
289 kD
mTOR
289 kD
p-AKT
60 kD
AKT
60 kD
PI3K
110 kD
GAPDH
36 kD
control
BSA
50
100
200
AOPP(μg/mL)
~289 kD
p-mTOR
~289 kD
mTOR
~110 kD
PI3K
~60 kD
p-AKT
~36 kD
GAPDH
~60 kD
AKT

Fig 3B

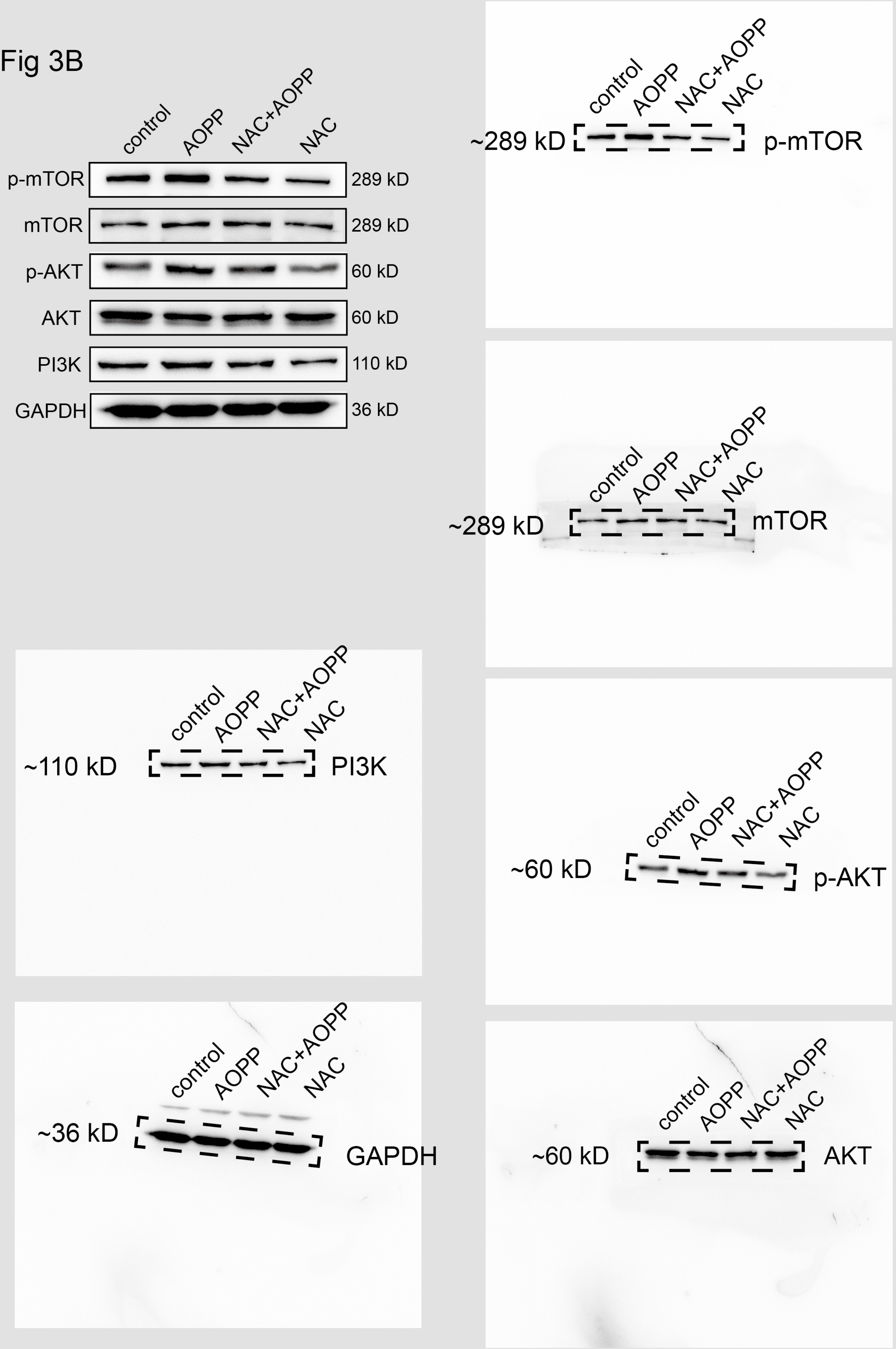
control
AOPP
NAC+AOPP
NAC
p-mTOR
289 kD
mTOR
289 kD
p-AKT
60 kD
AKT
60 kD
PI3K
110 kD
GAPDH
36 kD
~289 kD
p-mTOR
~289 kD
mTOR
~110 kD
PI3K
~60 kD
p-AKT
~36 kD
GAPDH
~60 kD
AKT

Fig 3B

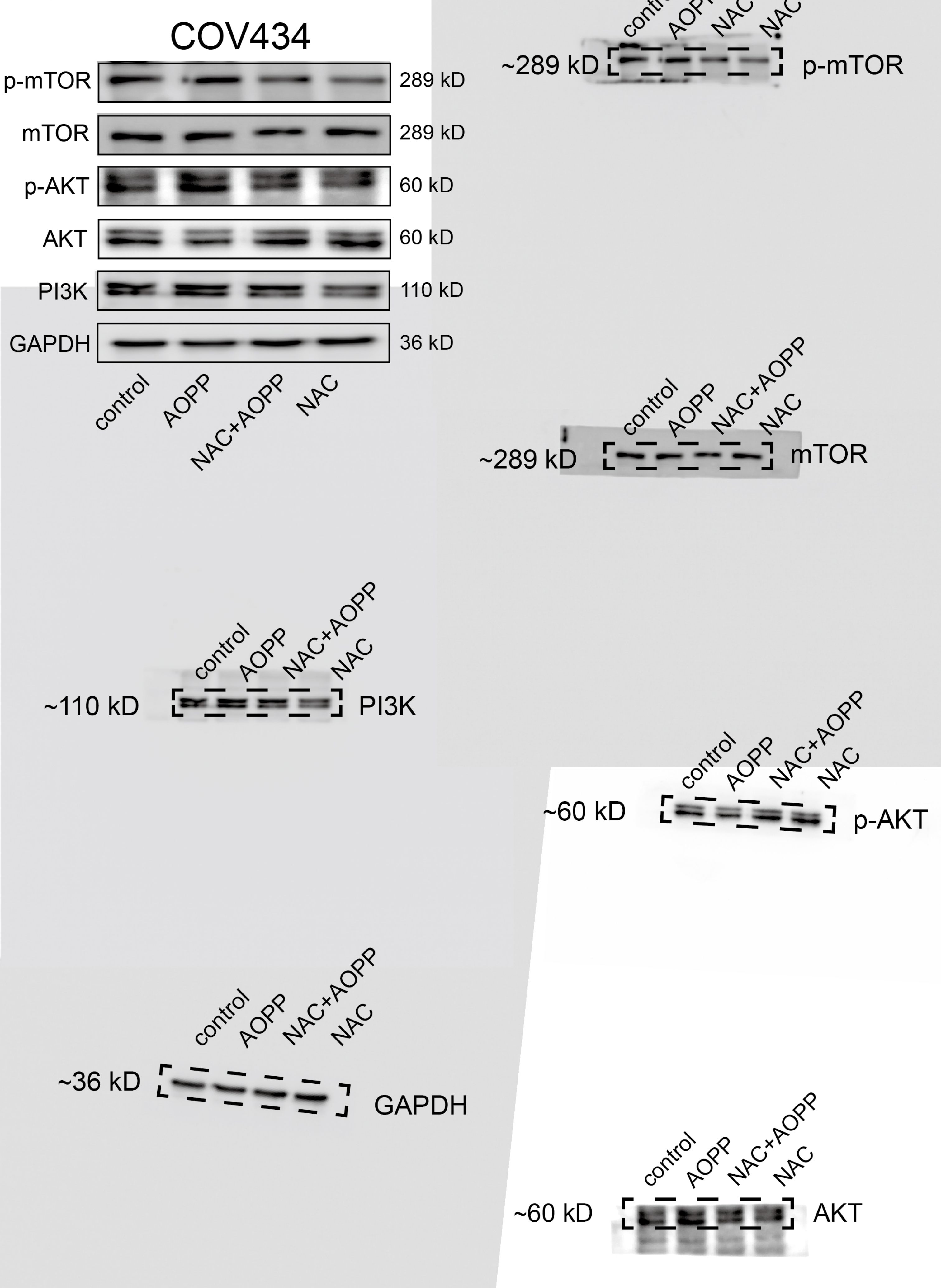

COV434
p-mTOR
mTOR
p-AKT
AKT
PI3K
GAPDH
289 kD
289 kD
60 kD
60 kD
110 kD
36 kD
control
AOPP
NAC+AOPP
NAC
~289 kD
p-mTOR
~289 kD
mTOR
~110 kD
PI3K
~60 kD
p-AKT
~36 kD
GAPDH
~60 kD
AKT

Fig 3C

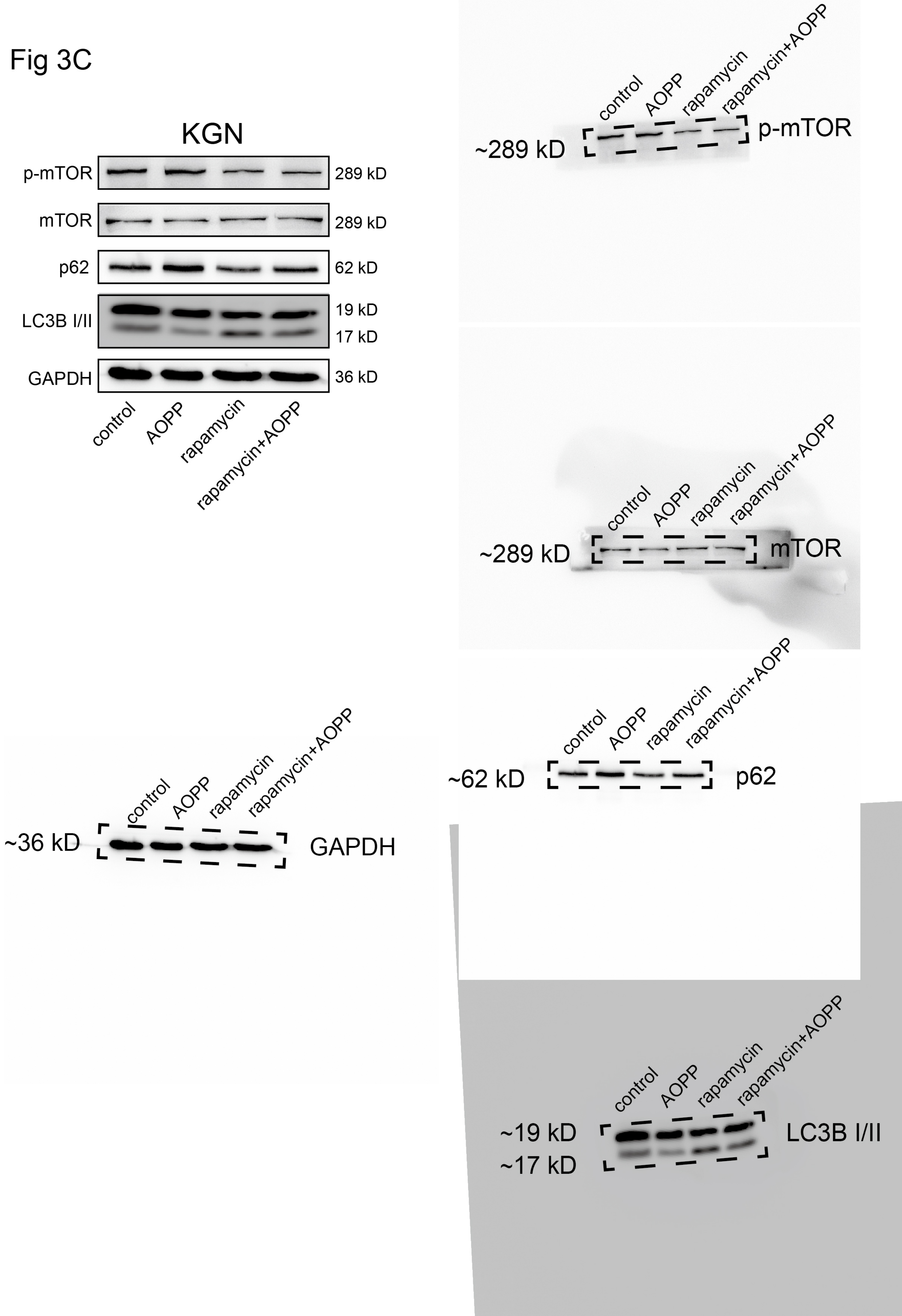
KGN
p-mTOR
289 kD
mTOR
289 kD
p62
62 kD
LC3B I/II
19 kD
17 kD
GAPDH
36 kD
control
AOPP
rapamycin
rapamycin+AOPP
~289 kD
p-mTOR
~289 kD
mTOR
~62 kD
p62
~36 kD
GAPDH
~19 kD
~17 kD
LC3B I/II

Fig 3C

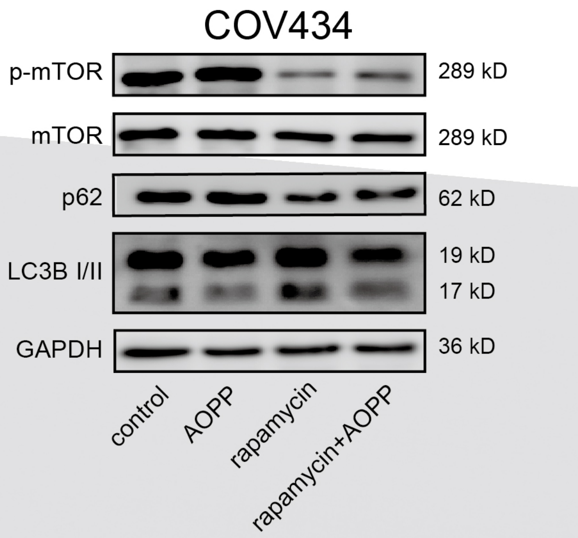
COV434
p-mTOR
mTOR
p62
LC3B I/II
GAPDH
289 kD
289 kD
62 kD
19 kD
17 kD
36 kD
control
AOPP
rapamycin
rapamycin+AOPP

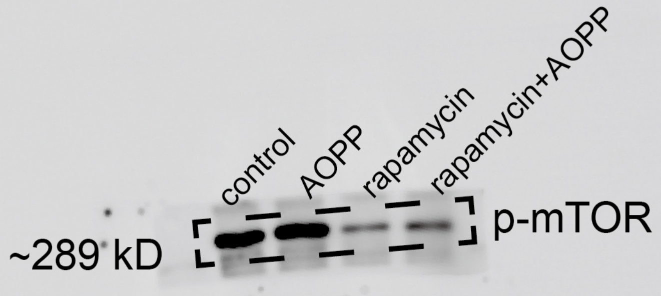
control
AOPP
rapamycin
rapamycin+AOPP
~289 kD
p-mTOR

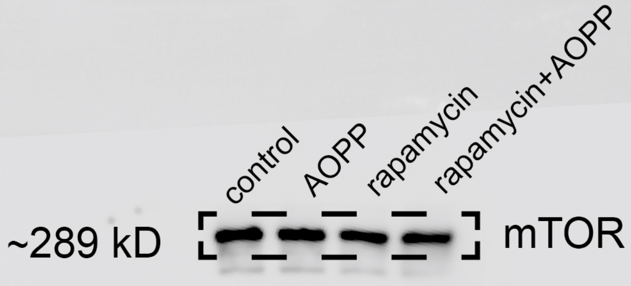
control
AOPP
rapamycin
rapamycin+AOPP
~289 kD
mTOR

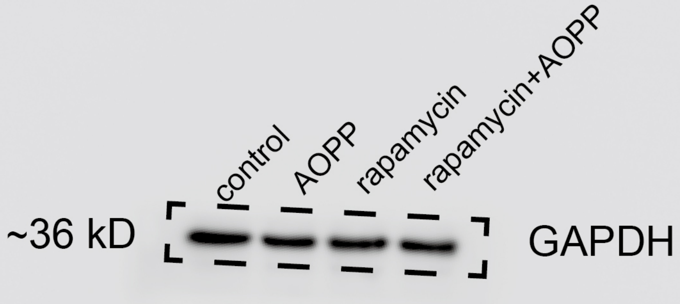
control
AOPP
rapamycin
rapamycin+AOPP
~36 kD
GAPDH

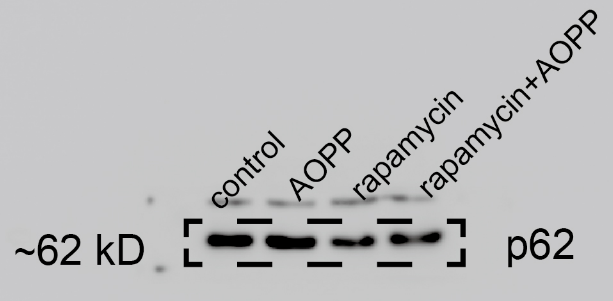
control
AOPP
rapamycin
rapamycin+AOPP
~62 kD
p62

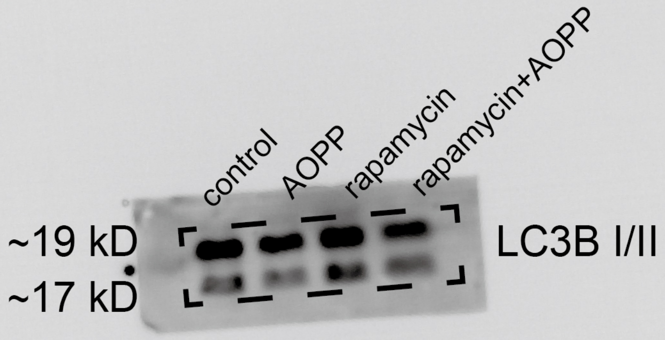
control
AOPP
rapamycin
rapamycin+AOPP
~19 kD
~17 kD
LC3B I/II

Fig 3D

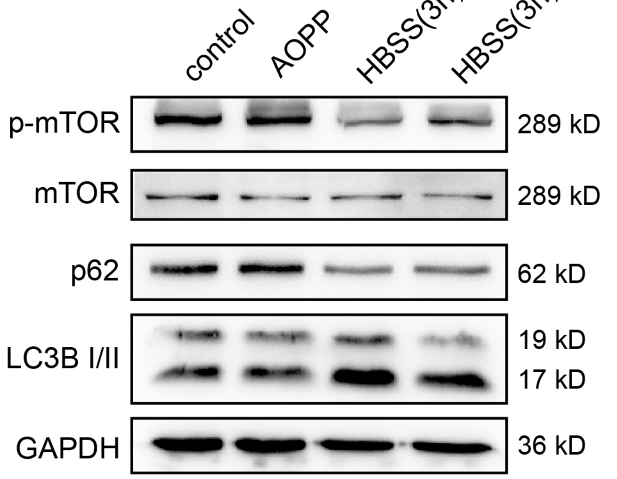
control
AOPP
HBSS(3h)
HBSS(3h)+AOPP
p-mTOR
289 kD
mTOR
289 kD
p62
62 kD
LC3B I/II
19 kD
17 kD
GAPDH
36 kD

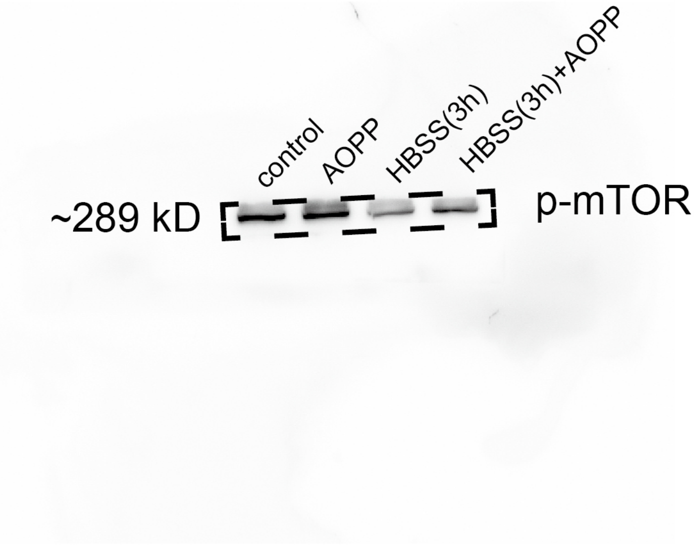
control
AOPP
HBSS(3h)
HBSS(3h)+AOPP
~289 kD
p-mTOR

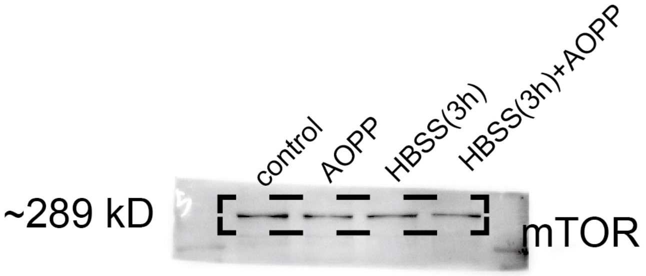
control
AOPP
HBSS(3h)
HBSS(3h)+AOPP
~289 kD
mTOR

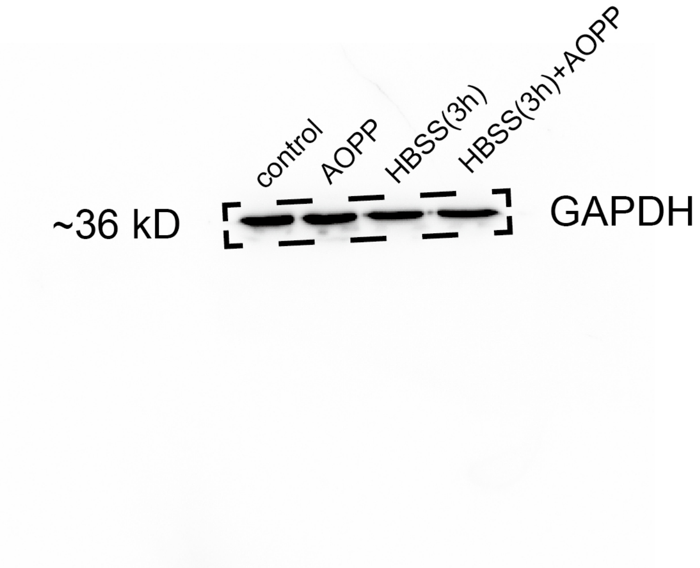
control
AOPP
HBSS(3h)
HBSS(3h)+AOPP
~36 kD
GAPDH

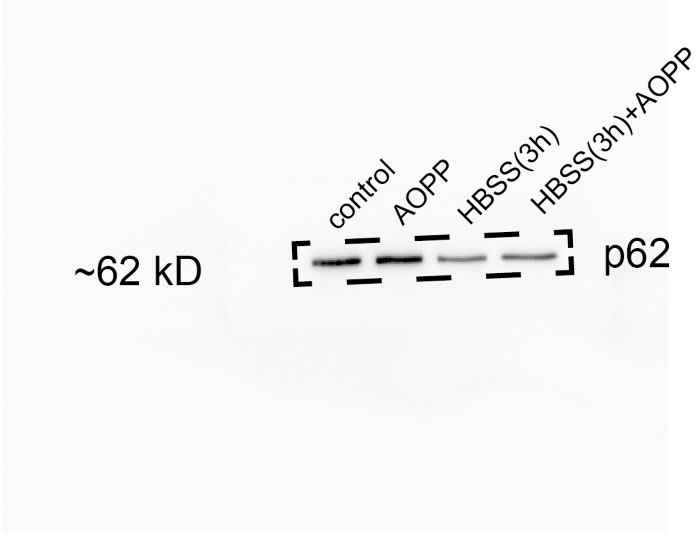
control
AOPP
HBSS(3h)
HBSS(3h)+AOPP
~62 kD
p62

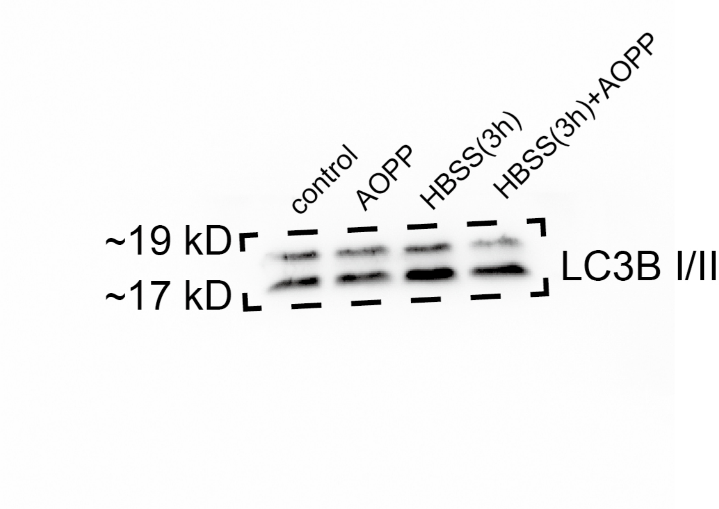
control
AOPP
HBSS(3h)
HBSS(3h)+AOPP
~19 kD
~17 kD
LC3B I/II

Fig 3D

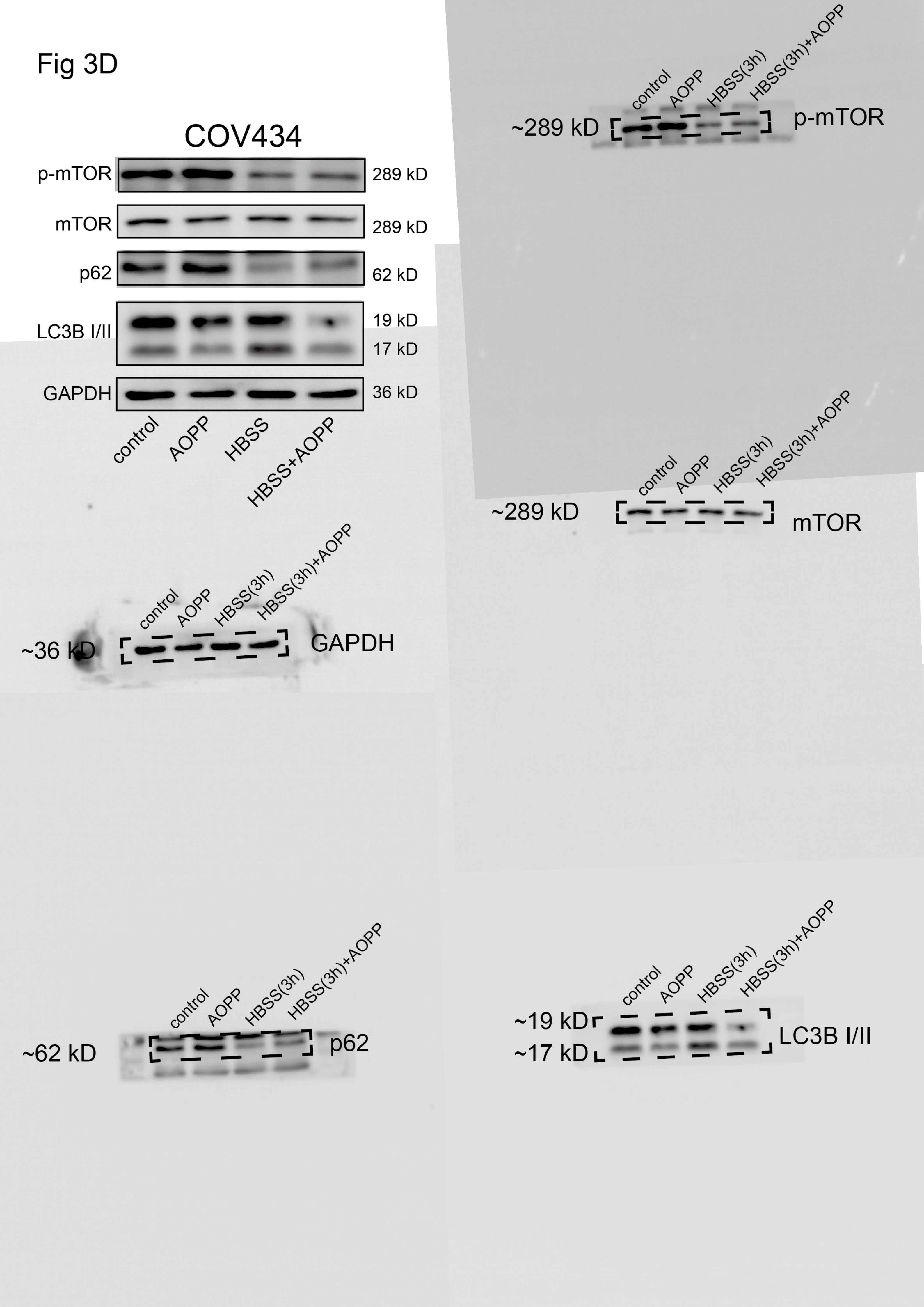
COV434
p-mTOR
289 kD
mTOR
289 kD
p62
62 kD
LC3B I/II
19 kD
17 kD
GAPDH
36 kD
control
AOPP
HBSS
HBSS+AOPP
control
AOPP
HBSS(3h)
HBSS(3h)+AOPP
~289 kD
p-mTOR
control
AOPP
HBSS(3h)
HBSS(3h)+AOPP
~289 kD
mTOR
control
AOPP
HBSS(3h)
HBSS(3h)+AOPP
~36 kD
GAPDH
control
AOPP
HBSS(3h)
HBSS(3h)+AOPP
~62 kD
p62
control
AOPP
HBSS(3h)
HBSS(3h)+AOPP
~19 kD
~17 kD
LC3B I/II

Fig 4A

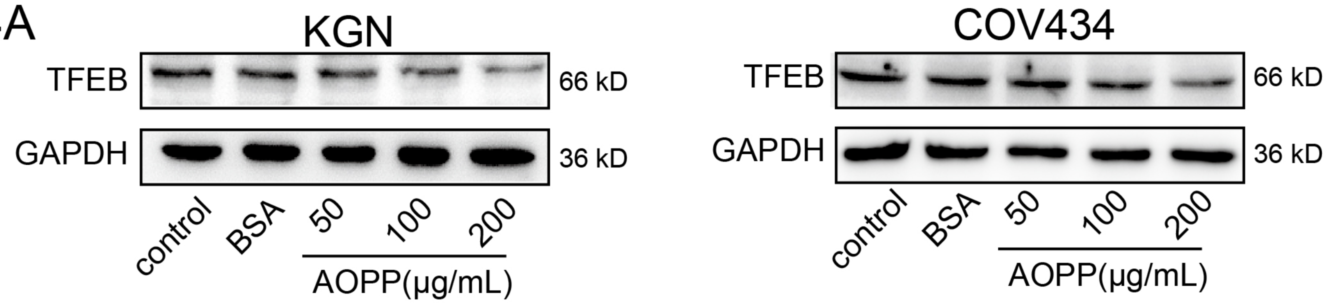
KGN
TFEB
66 kD
GAPDH
36 kD
control
BSA
50
100
200
AOPP(μg/mL)
COV434
TFEB
66 kD
GAPDH
36 kD
control
BSA
50
100
200
AOPP(μg/mL)

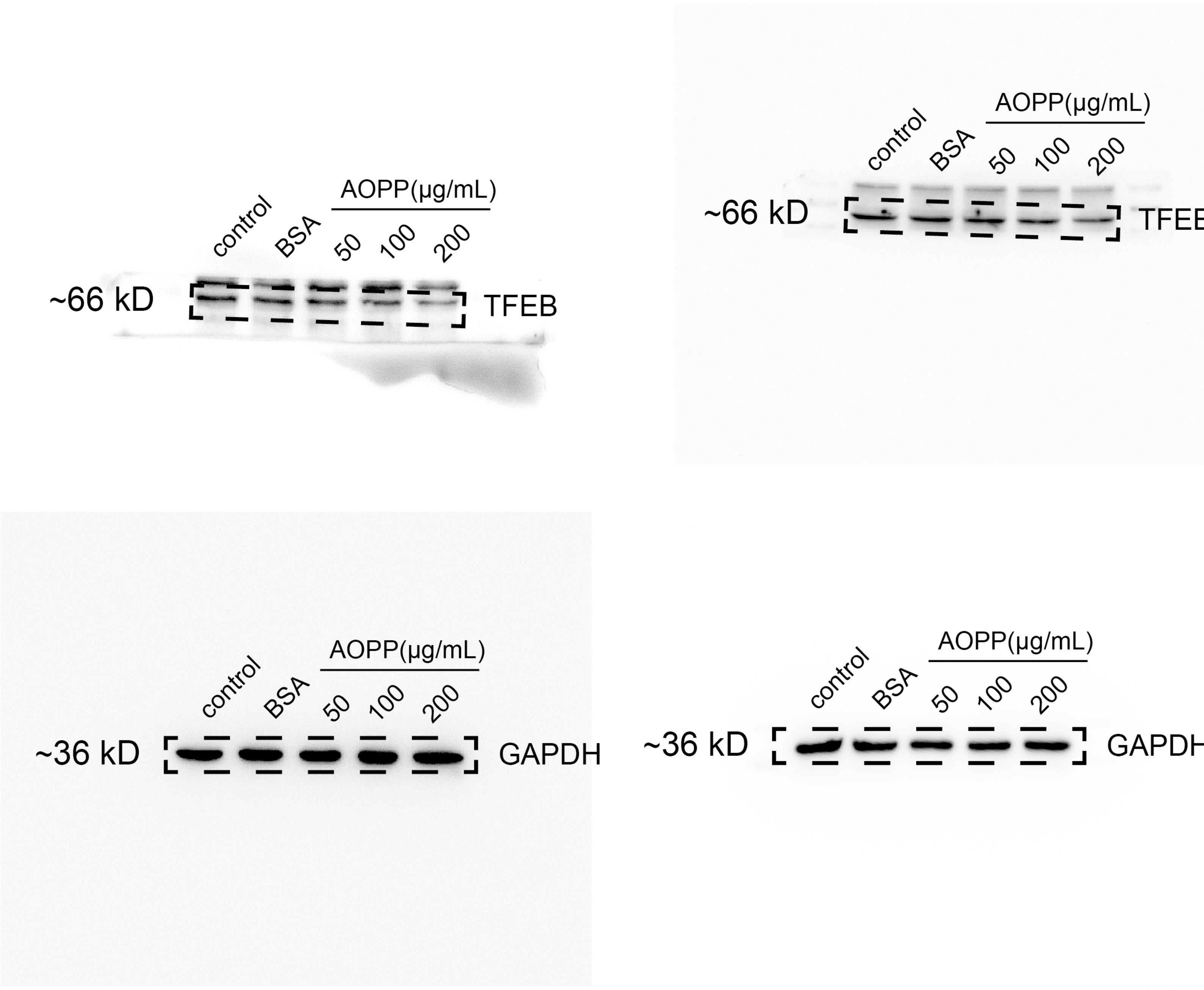
control
BSA
AOPP(μg/mL)
50
100
200
~66 kD
TFEB
control
BSA
AOPP(μg/mL)
50
100
200
~66 kD
TFEB
control
BSA
AOPP(μg/mL)
50
100
200
~36 kD
GAPDH
control
BSA
AOPP(μg/mL)
50
100
200
~36 kD
GAPDH

Fig 4B

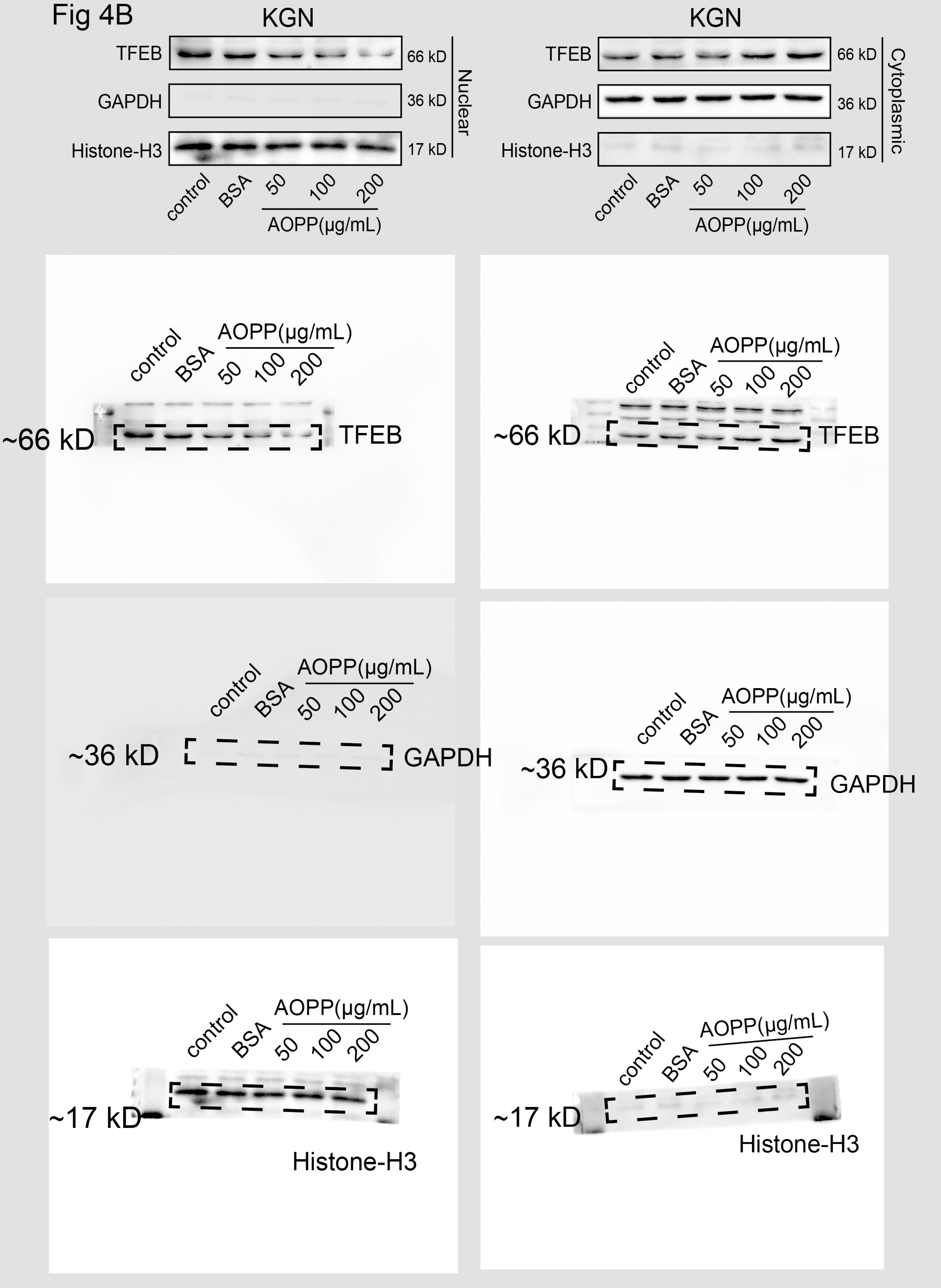
KGN
TFEB
GAPDH
Histone-H3
66 kD
36 kD
17 kD
Nuclear
Cytoplasmic
control
BSA
50
100
200
AOPP(μg/mL)
~66 kD
TFEB
~36 kD
GAPDH
~17 kD
Histone-H3

Fig 4B

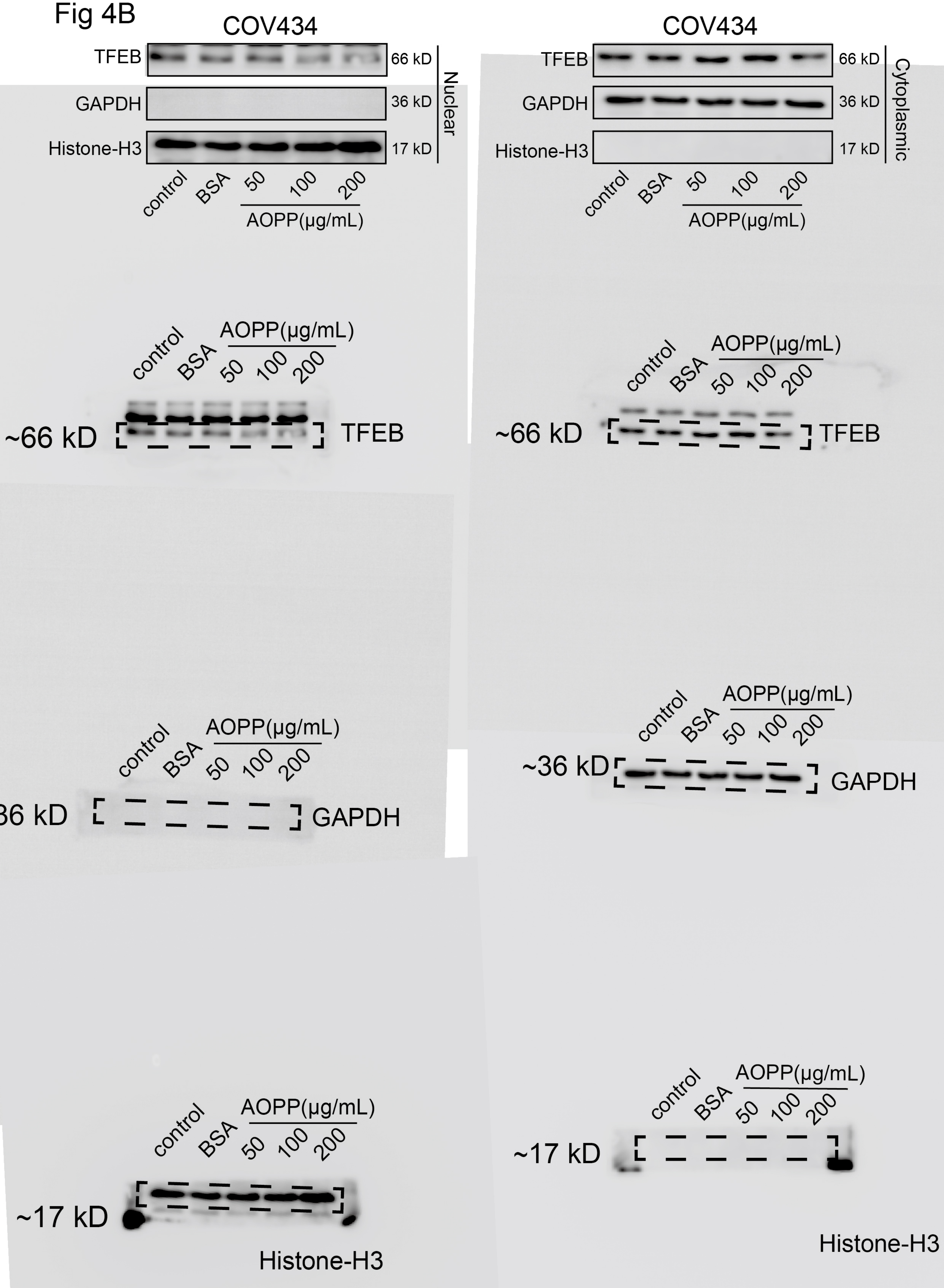

COV434
TFEB
66 kD
GAPDH
36 kD
Histone-H3
17 kD
Nuclear
control
BSA
50
100
200
AOPP(μg/mL)
COV434
TFEB
66 kD
GAPDH
36 kD
Histone-H3
17 kD
Cytoplasmic
control
BSA
50
100
200
AOPP(μg/mL)
control
BSA
AOPP(μg/mL)
50
100
200
~66 kD
TFEB
control
BSA
AOPP(μg/mL)
50
100
200
~66 kD
TFEB
control
BSA
AOPP(μg/mL)
50
100
200
36 kD
GAPDH
control
BSA
AOPP(μg/mL)
50
100
200
~36 kD
GAPDH
control
BSA
AOPP(μg/mL)
50
100
200
~17 kD
Histone-H3
control
BSA
AOPP(μg/mL)
50
100
200
~17 kD
Histone-H3

Fig 4C

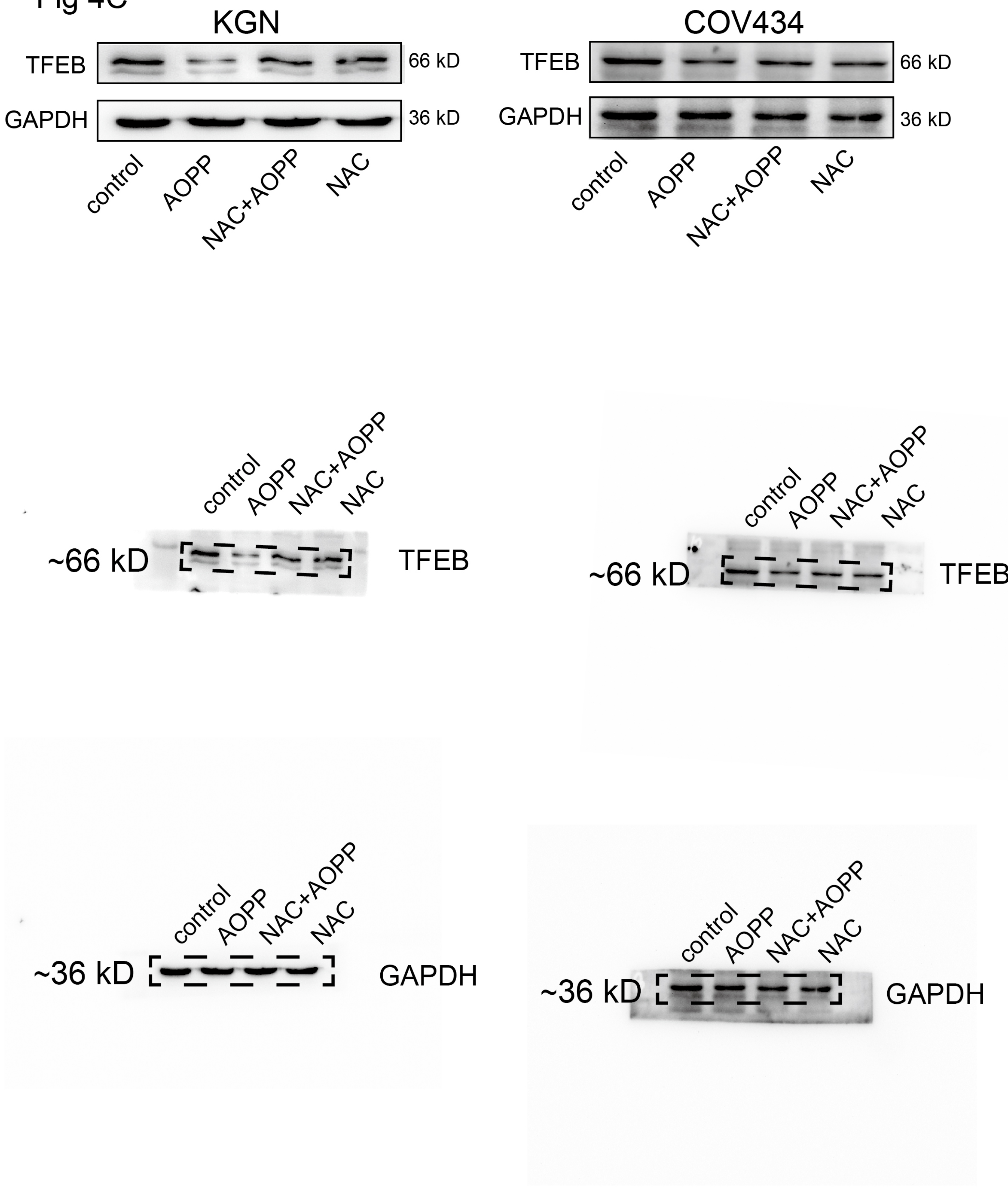
KGN
COV434
TFEB
GAPDH
66 kD
36 kD
control
AOPP
NAC+AOPP
NAC
~66 kD
~36 kD

Fig 4D

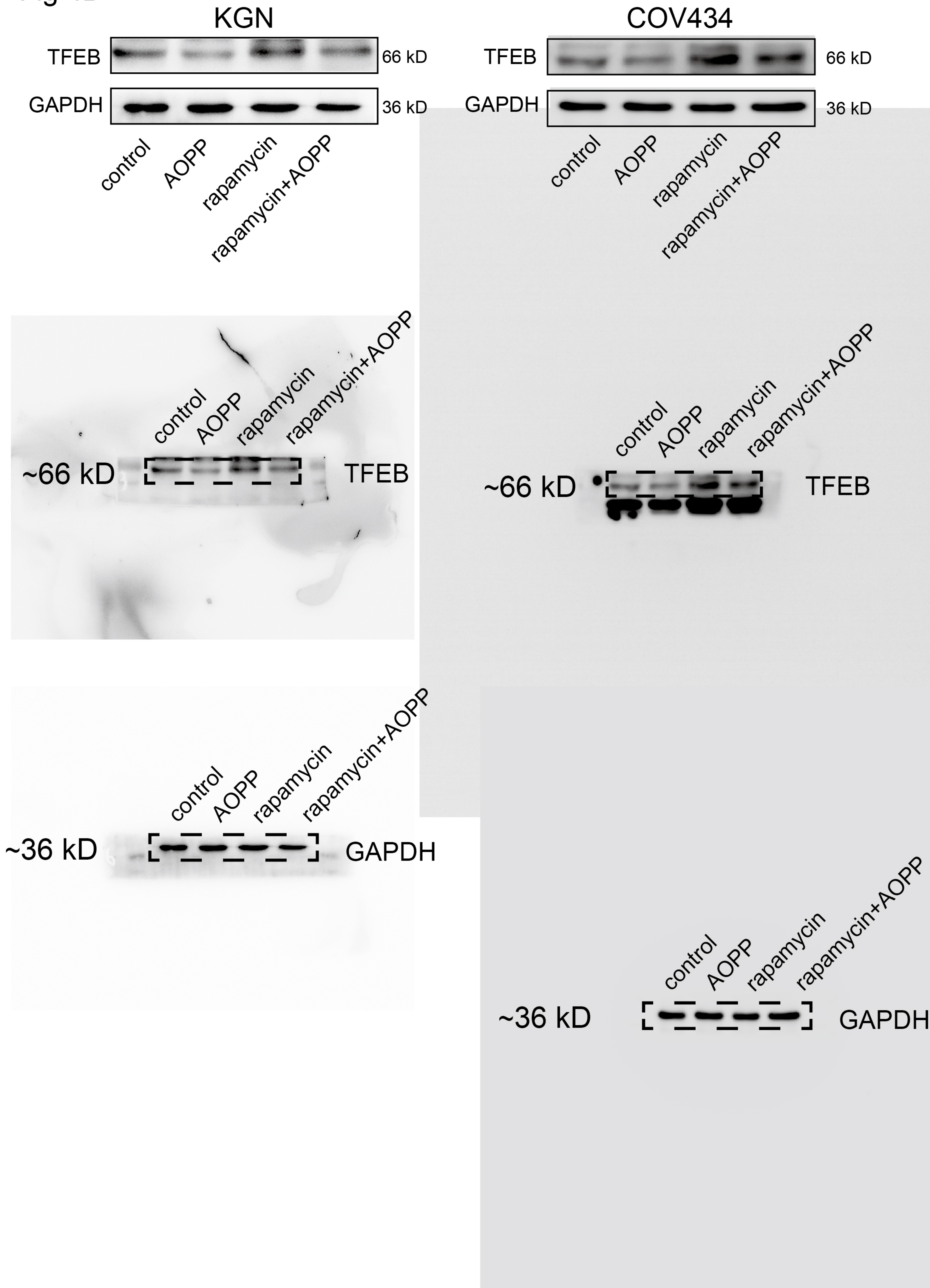

KGN
TFEB
66 kD
GAPDH
36 kD
control
AOPP
rapamycin
rapamycin+AOPP
COV434
TFEB
66 kD
GAPDH
36 kD
control
AOPP
rapamycin
rapamycin+AOPP
control
AOPP
rapamycin
rapamycin+AOPP
~66 kD
TFEB
control
AOPP
rapamycin
rapamycin+AOPP
~66 kD
TFEB
control
AOPP
rapamycin
rapamycin+AOPP
~36 kD
GAPDH
control
AOPP
rapamycin
rapamycin+AOPP
~36 kD
GAPDH

Fig 4E

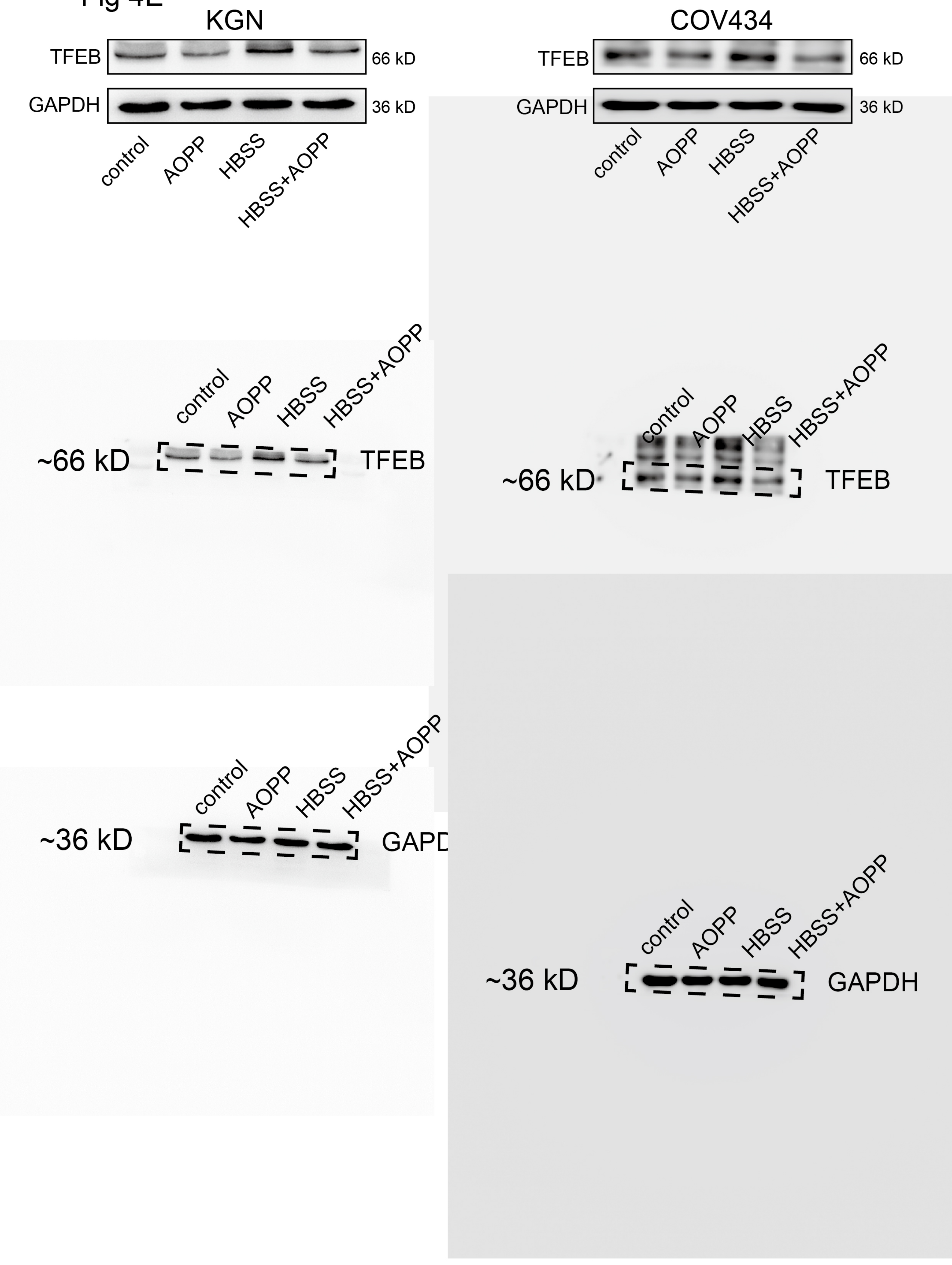

KGN
TFEB
66 kD
GAPDH
36 kD
control
AOPP
HBSS
HBSS+AOPP
COV434
TFEB
66 kD
GAPDH
36 kD
control
AOPP
HBSS
HBSS+AOPP
control
AOPP
HBSS
HBSS+AOPP
~66 kD
TFEB
control
AOPP
HBSS
HBSS+AOPP
~66 kD
TFEB
control
AOPP
HBSS
HBSS+AOPP
~36 kD
GAPD
control
AOPP
HBSS
HBSS+AOPP
~36 kD
GAPDH

Fig 5B

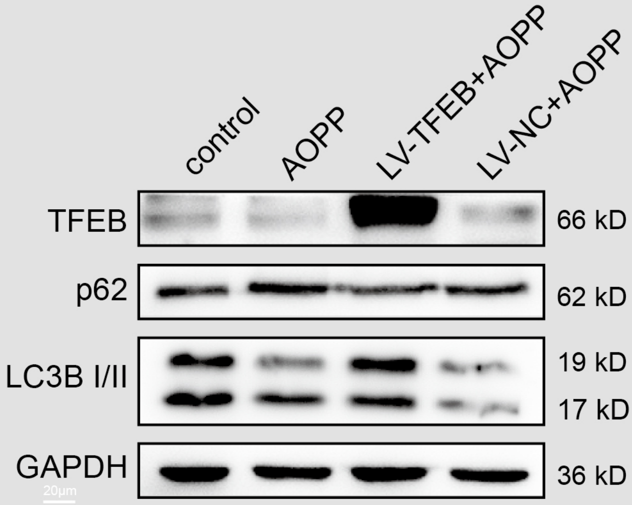
control
AOPP
LV-TFEB+AOPP
LV-NC+AOPP
TFEB
66 kD
p62
62 kD
LC3B I/II
19 kD
17 kD
GAPDH
36 kD

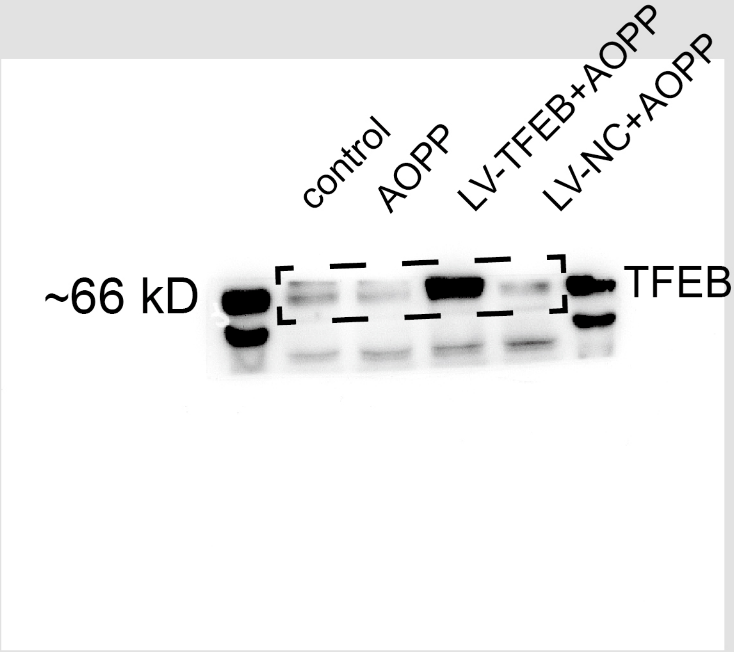
control
AOPP
LV-TFEB+AOPP
LV-NC+AOPP
~66 kD
TFEB

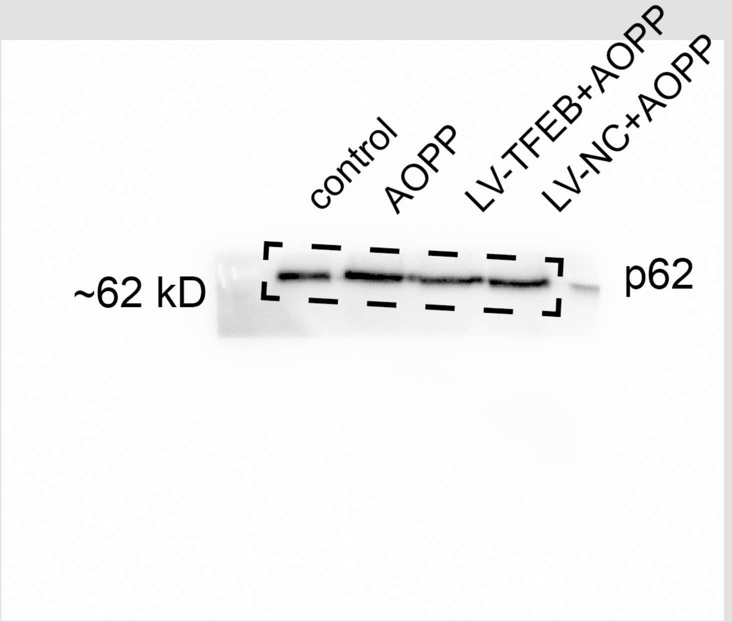
control
AOPP
LV-TFEB+AOPP
LV-NC+AOPP
~62 kD
p62

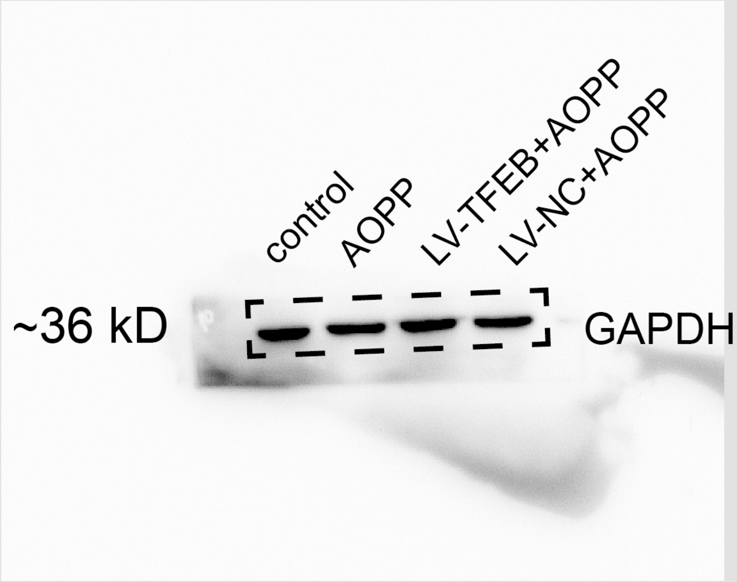
control
AOPP
LV-TFEB+AOPP
LV-NC+AOPP
~36 kD
GAPDH

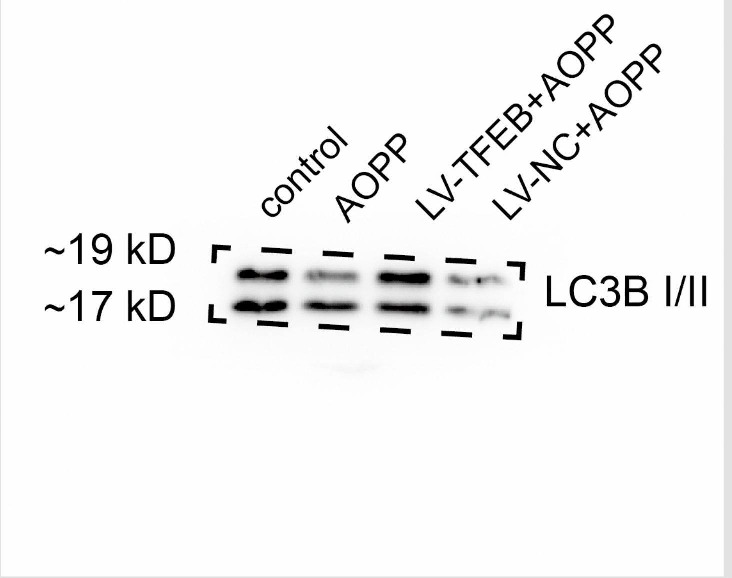
control
AOPP
LV-TFEB+AOPP
LV-NC+AOPP
~19 kD
~17 kD
LC3B I/II

Fig 5B

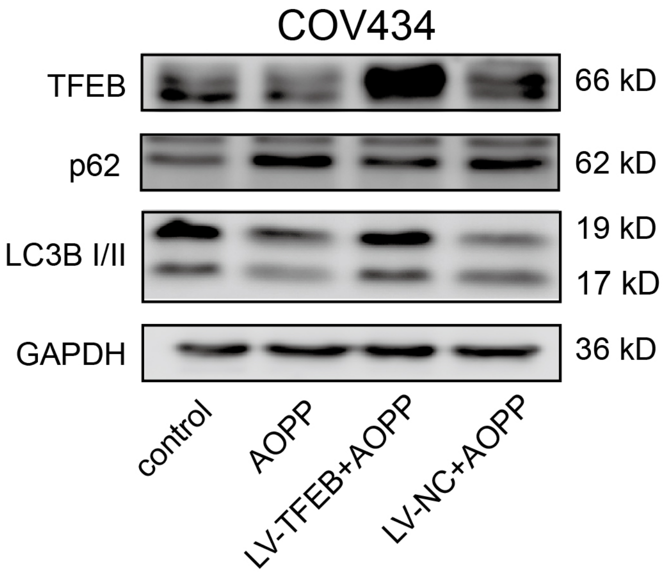
COV434
TFEB
66 kD
p62
62 kD
LC3B I/II
19 kD
17 kD
GAPDH
36 kD
control
AOPP
LV-TFEB+AOPP
LV-NC+AOPP

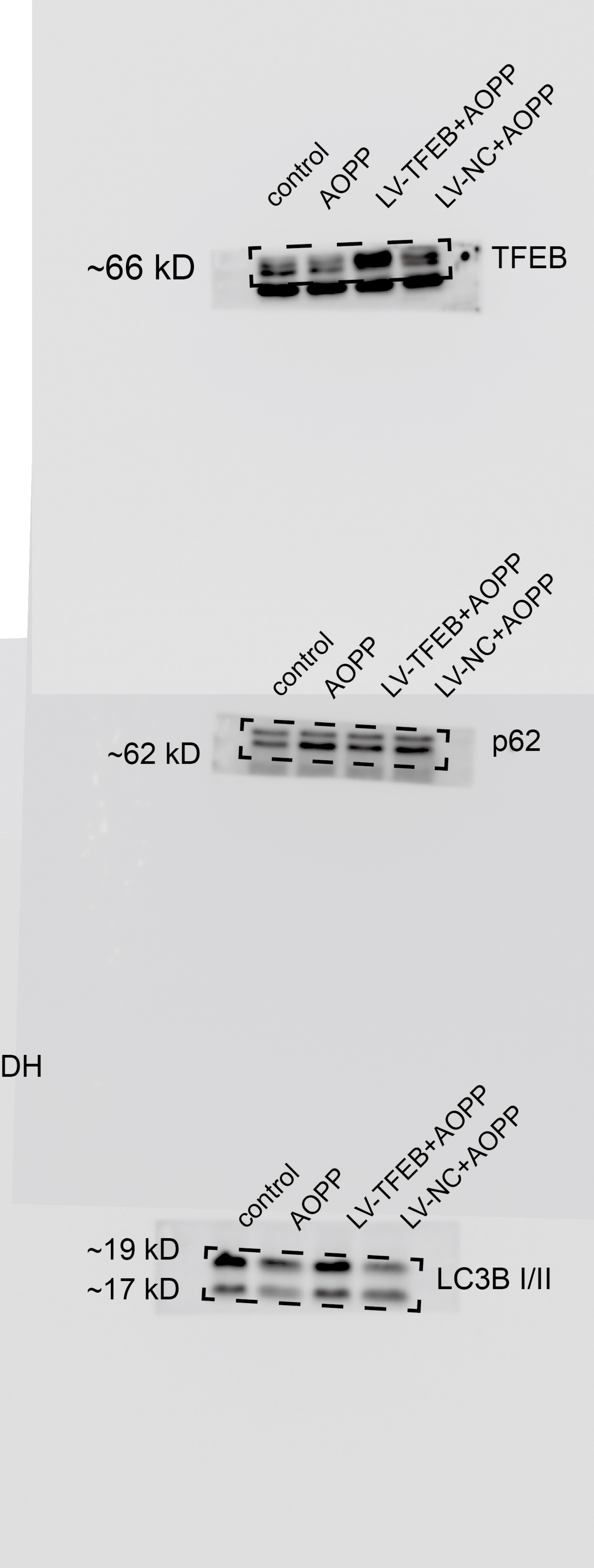
control
AOPP
LV-TFEB+AOPP
LV-NC+AOPP
~66 kD
TFEB
control
AOPP
LV-TFEB+AOPP
LV-NC+AOPP
~62 kD
p62
control
AOPP
LV-TFEB+AOPP
LV-NC+AOPP
~19 kD
~17 kD
LC3B I/II

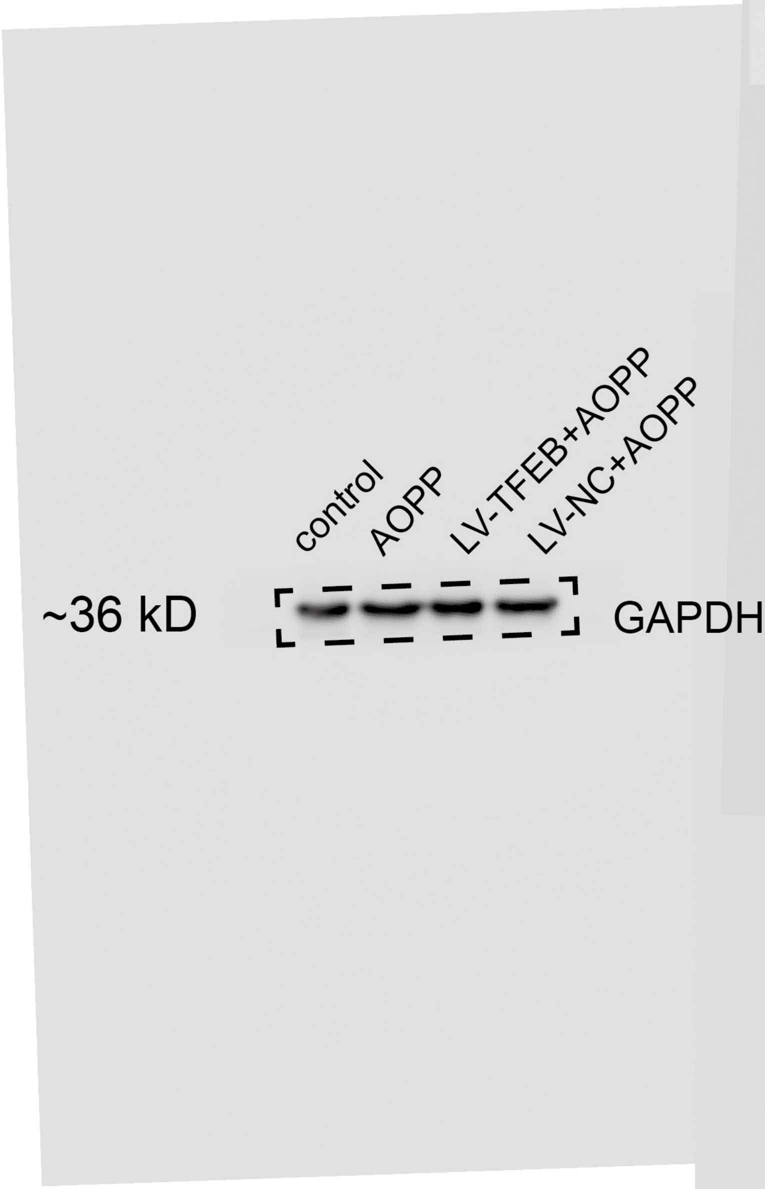
control
AOPP
LV-TFEB+AOPP
LV-NC+AOPP
~36 kD
GAPDH

Fig 6B

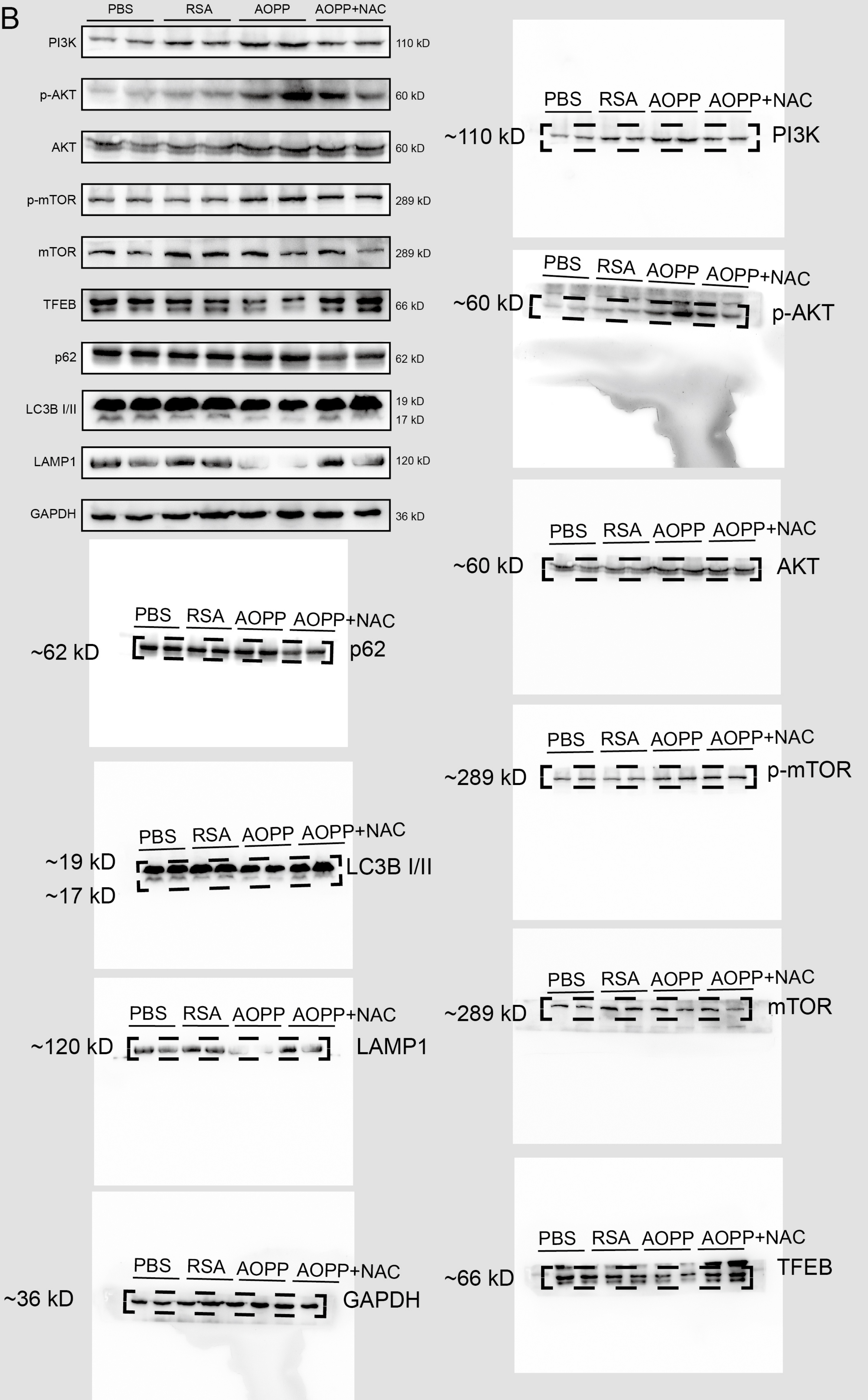

PBS
RSA
AOPP
AOPP+NAC
PI3K
110 kD
p-AKT
60 kD
AKT
60 kD
p-mTOR
289 kD
mTOR
289 kD
TFEB
66 kD
p62
62 kD
LC3B I/II
19 kD
17 kD
LAMP1
120 kD
GAPDH
36 kD
~110 kD
~60 kD
~62 kD
~289 kD
~19 kD
~17 kD
~120 kD
~66 kD
~36 kD
